# Erythrocyte Concentrates Recovered from Under-Collected Whole Blood: Experimental and Clinical Results

**DOI:** 10.1371/journal.pone.0117928

**Published:** 2015-02-23

**Authors:** Wen-Biao Liang, Ming-Hua Guo, En-Yong Fan, Jing-Jing Zhang, Min-Hui Wu, Yan-Chun Liu, Li Cai, Zheng-Gang Li, Bing Huang, Peng Wei, Jun Sun, Yi Zhu

**Affiliations:** 1 Research Department of Jiangsu Province Blood Center, Nanjing, Jiangsu Province, P. R. China; 2 Quality Management Department of Yangzhou Red-Cross Blood Bank, Yangzhou, Jiangsu Province, P. R. China; 3 Department of General Surgery, First Affiliated Hospital, Nanjing Medical University, Nanjing, Jiangsu Province, P. R. China; 4 Preparation Division of Jiangsu Province Blood Center, Nanjing, Jiangsu Province, P. R. China; 5 Management Department of Jiangsu Province Blood Center, Nanjing, Jiangsu Province, P. R. China; Royal College of Surgeons, IRELAND

## Abstract

**Background:**

Although periodic blood shortages are widespread in major Chinese cities, approximately 1x10^5^ U of whole blood are discarded yearly because of under-collection. To reduce the wastage of acid citrate dextrose solution B (ACD-B) anticoagulated under-collected whole blood (UC-WB), this study was performed to elucidate the effect of extracellular pH and holding time on erythrocyte quality. Mannitol-adenine-phosphate (MAP) erythrocyte concentrates (UC-RBCs) were prepared with UC-WB to assess the safety and efficacy of this component.

**Methods:**

The effect of the different extracellular pH levels and storage times on erythrocytes was assessed by fluorescent probes, SDS-PAGE electrophoresis, electron microscopy and spectroscopy. In vitro properties of 34 UC-RBCs that were prepared with UC-WB at different times after collection were analyzed and compared to normal RBCs during 35 days of storage. The results of transfusion with UC-RBCs and the incidence of adverse reactions in 49 patients were determined.

**Results:**

1) Low extracellular pH levels and long storage time induced increases in RBC fluorescence polarization and mean microviscosity, changes in membrane fluidity, band 1, 2 and 3 protein expression, and erythrocyte morphology. 2) During storage for 35 days, difference in between-subjects effects of K^+^, hemolysis and supernatant erythrocyte membrane protein (EMP) were statistically significant (P = 0.041, 0.007 and 0.002, respectively), while the differences between these parameters in the 4 h group and comparable controls were less significant. 3) Clinical data from 49 patients confirmed that transfusions with UC-RBCs were satisfactory with no adverse reactions.

**Conclusion:**

These results suggest that it is feasible to prepare RBCs with ACD-B anticoagulated UC-WB at a minimum of 66% volume of the labeled collection. It was effective and safe to transfuse the UC-RBCs prepared within 4 h after collection and stored within 7 days. The use of UC-WB would be a welcome addition to limited blood resources in China.

**Trial Registration:**

Chinese Clinical Trial Registry ChiCTR-TRC-13003967

## Introduction

Over the past 15 years, the supply of blood for transfusion in China has attained an average annual growth of 12.6% but is still unable to meet the clinical need. Despite intensive efforts to increase the number of blood donors, the marginal supply remains unchanged; blood shortages have changed from seasonal and/or structural a few years ago to conventional today in major Chinese cities.

However, large quantities of allogeneic whole blood units are discarded because of an insufficient collected volume. The percentage of units with unsatisfactory volumes varies among regions and blood banks, according to data publicly reported in the Chinese literature; a conservative assumption states that the percentage of under-collected units ranges from 0.5% to 1.0% of all collections and therefore at least several million liters of whole blood are discarded yearly in China because of under-collection. In the USA, this percentage is between 2.5% and 3.5% of all blood collections [1, 2].

At present, there is limited information regarding the in vitro and in vivo behavior of under-collected whole blood, in which an acceptable volume for the clinical application of under-collected units differs according to different models. For the collection of 450 ml of whole blood, Button et al. demonstrated that 400 g blood samples collected in ACD solution or 300 g in CPD anticoagulant were acceptable. Davey et al. [1] chose 275 ml as the minimum clinically useful volume of blood in CPDA-1 [3]. Joseph et al. showed that donation volumes of between 180 and 211 ml in CP2D-A could be transfused with a shortened expiry of 21 days [2]. However, for the component prepared with UC-WB, only one report exists, in which Weisbach et al. determined the quality of RBCs stored in PAGGS-M additive solution prepared from 150 to 300 ml under-collected whole blood in CPD solution [4]. To date, we are unaware of any reports in the literature that could inform guidelines on how to treat inadequately filled units of ACD-B anticoagulated whole blood to prepare MAP erythrocyte concentrates.

Although the ACD-B anticoagulant was gradually replaced by CPD/CPDA solution in whole blood collection in Europe and North America because of the “acid load” imposed by excess citric acid [5, 6], it remains the most commonly used in blood banking practice in China. However, UC-WB erythrocytes could be impaired by an acidic intracellular environment, and the quality of stored UC-WB cannot be guaranteed at the end of whole blood storage time. To explore the basis for the clinical use of ACD-B anticoagulated UC-WB (minimum 66% volume of the intended collection), and to determine the feasibility of transfusing the MAP erythrocyte concentrates prepared with these under-collected units. We designed the first experiment to study the relationship between the extracellular pH environment (an approximate representation of WB collected volume), the holding time and the RBC quality. Then, we analyzed the properties of 34 UC-RBCs in vitro that were prepared with UC-WB >66% volume of the labeled collection at different holding times compared to those of normal RBCs during 35 days of storage. Finally, we examined the safety and efficacy of the transfusion of UC-RBCs in 49 patients. These results provide experimental evidence for the clinical use of UC-RBCs and the protocol for this trial and supporting CONSORT checklist are available as supporting information (see S1 CONSORT Checklist and S1 Protocol).

## Materials and Methods

### The effect of different extracellular pH environments and holding times on RBC quality

A pool-and-split study design was used to investigate the effects, as follows: six healthy blood donors with group A and RhD-positive blood were randomly selected, and 5 ml venous blood was simultaneously collected from these donors. Immediately after collection, these matched WB were washed, pooled, mixed, and aliquoted to make five identical RBC units. One unit from each pool was suspended in ACD-B anticoagulant pH (5.0), and four additional anticoagulant solutions that were identical in composition to ACD-B but with different pH levels (5.6, 6.2, 6.8 and 7.4) according to the addition of NaOH. The proportion of red blood cells to anticoagulant was 4:6. Then, all samples in were stored at 4°C in capped tubes. The membrane fluidity, membrane protein, morphology of the erythrocyte membrane and concentration of FHb at 4, 8 and 24 h were measured by fluorescent probes, SDS-PAGE electrophoresis, electron microscopy and spectroscopy, respectively.

For membrane fluidity, erythrocyte ghosts were prepared according to the method of Steck and Kant [7]. Fluorescence depolarization was measured as described previously by Shinitzky and Barenholz [8]. Briefly, after erythrocyte membranes were incubated for 30 min at room temperature with 1,6-diphenylhexa-1,3,5-triene (DPH; Sigma) in tetrahydrofuran to a final concentration of 50 μg/ml for protein and 1 × 10^-6^mol/L for fluorescence labeling, fluorescence-polarization measurements were performed on a RF-540 luminescence spectrometer (Shimadzu). Excitation was set at 362 nm, and emission was detected at 432 nm using a 10 nm band-pass on both light-paths. The degree of fluorescence polarization (P) was calculated according to the following equations:

$$\begin{array}{l} P=\text{(Ivv}\text{-GIvh)}/\text{(Ivv}+\text{GIvh)}\\ \eta =\text{2}P\text{/(0}.46-P\text{)}\\ \text{G}=\text{Ihv}\text{/Ihh,} \end{array}$$

where *I*_vv_ and *I*_vh_ represent for the fluorescence intensities recorded with the analyzing polarizer oriented parallel with the plane of the excitation beam and perpendicular to it, respectively. *I*_hh_ was the intensity of emitted light when the two analyzing polarizer orientations were both horizontal. *I*_hv_ was the intensity of emission light when the emission polarizer was horizontal and the excitative polarizer was vertical. *G* denoted a correction factor.

For electron microscopy, samples were fixed in suspension in 0.5 M cacodylate buffer (pH 7.4), which contained 2.5% glutaraldehyde. The material was post-fixed with OsO_4_, dehydrated and embedded in Epon. Sections were stained on the grids with uranyl acetate and lead citrate, and observations were carried out on a JEM-1010 transmission electron microscope (JEOL). SDS-PAGE analysis was performed according to conventional techniques. FHb levels were tested spectrophotometrically by the cyanomethemoglobin method with Microlab 300 (Vital Scientific, Netherlands).

### The preparation of MAP RBCs from ACD-B anticoagulated UC-WB

Units of UC-WB that were more than 66% volume of the intended collection were selected to prepare MAP UC-RBCs. Blood components were investigated following manual preparation in a conventional triple or quadruple bag system. Briefly, after centrifugation at 3500 g for 15 min at 4°C with slow braking, the plasma and buffy coat were first removed in the transfer bag. Then, the packed red blood cells were weighed, and an appropriate volume of MAP additive solution was added in a sterile manner to the packed RBC at a ratio of 1:2 (v/v). The units were mixed well, stored under standard blood banking conditions at 4°C, and were sampled aseptically on days 0, 7, 14, 21, 28, and 35 of storage.

### In vitro properties of UC-RBCs

After gentle mixing by inversion, samples from stored units were collected by a sterile sampling procedure in a biological safety cabinet. The pH values were measured at ambient temperature (26°C) using a pH meter. Counts of total hemoglobin (Hb), hematocrit (Hct), red cells, leucocytes, and platelets, as well as the mean corpuscular volume, were performed on a Sysmex XT-1800 cell counter (Sysmex, Japan). The osmotic fragility of erythrocytes was determined by the brine osmosis method. Supernatant sodium and potassium were measured with the Easylyte Plus K^+^/Na^+^/Cl^−^ analyzer (MEDICA, Bedford, Massachusetts, USA). The levels of 2,3-diphosphoglycerate acid (2,3-DPG, R&D, Minneapolis, MN, USA) and EMP (Cusabio, Wuhan, China) were determined using commercial kits according to the recommended protocol.

Hemolysis was measured by determining supernatant Hb levels with conversion to percent hemolysis using total Hb and Hct levels [9]. Briefly, free Hb was determined by absorbance measurements of cell supernatants at 510 nm by a spectrophotometer, and hemolysis was expressed as a percentage of total Hb present in RBCs after a correction for the Hct. The rate of hemolysis was calculated using the following formula:

Hemolysis (%)=[100 - Hct (%)]× Free Hb (mg/dL) / Total Hb (mg/dL)× 100

### Clinical assessment of UC-RBCs

All ongoing and related trials for this intervention have been registered: this study was part of a clinical trial (Controlled Trials number: ChiCTR-TRC-13003967, Chinese Clinical Trial Registry) to assess the safety and efficiency of UC-RBCs. Ethics approval was obtained for the study from the Medical Ethics Committee of Jiangsu Province Blood Center (ref: 2012001) on the 15^th^ March 2012. All participants involved in our study at the First Affiliated Hospital, the Second Affiliated Hospital, Nanjing Medical University and the Nanjing General Hospital of Nanjing Military Command gave written informed consent.

**Fig 1:**
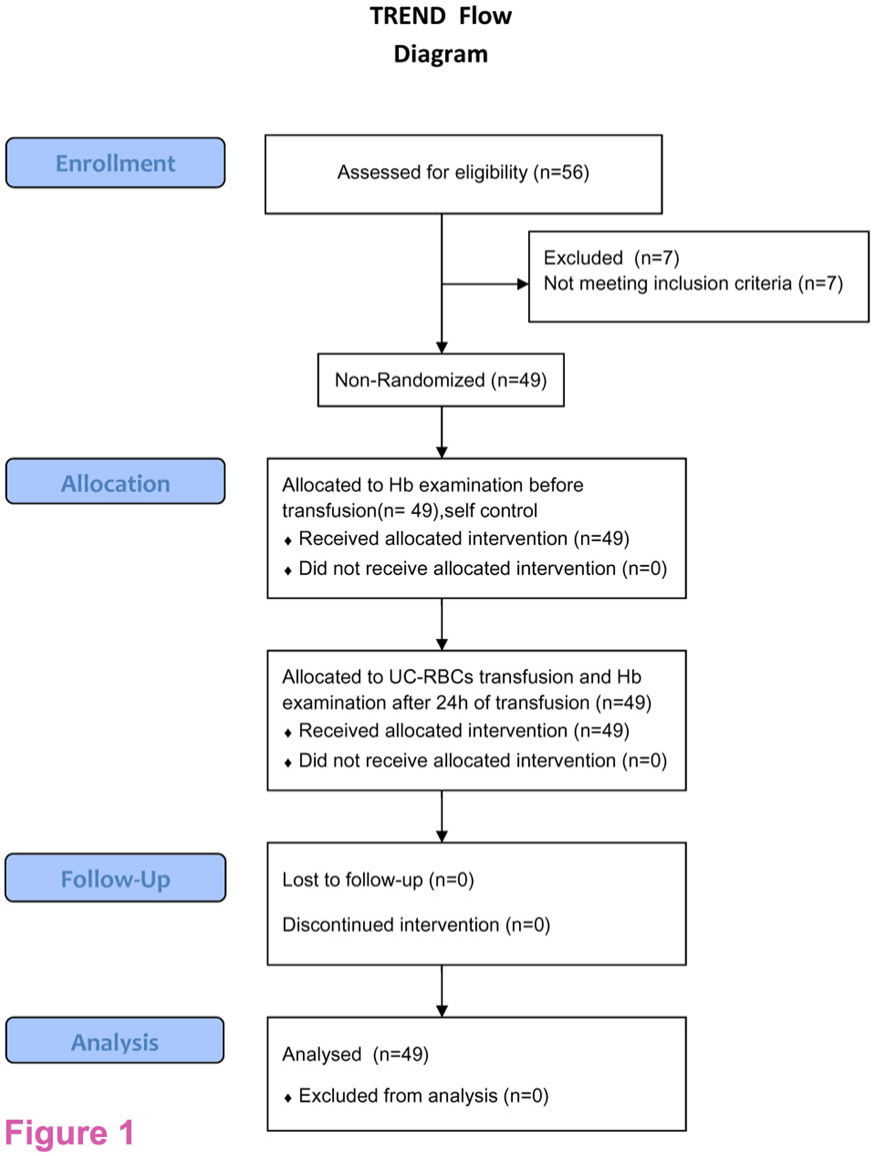
Flow chart of patient involvement in this clinical trial. This trial enrolled 56 participants, in which seven patients were excluded as they did not meet inclusion criteria.

The study enrolled 56 patients who were older than 18 years of age and had indications for transfusion, as defined by the technical specifications of the clinical blood transfusion department: the patients transfused were those who underwent preplanned surgeries, or those whose Hb values were <70 g/L, were an internal medicine patient who suffered from one or more clinical symptoms of anemia, such as pallor, giddiness, shortness of breath, or palpitations. Important exclusion criteria were active bleeding, hemolytic disease, autoimmune diseases and serious systemic disease or complications. For each case, the patient had at least 1 U of UC-RBCs transfused, which were prepared within 4 h after collection and stored for less than 1 week. The adverse reactions as referenced by the European Hemovigilance Network (EHN), were observed and followed-up during and within 180 days of transfusion, and changes in the Hb level of each patient before and 24 h after the transfusion were assessed. The flowchart of patient involvement in this non-randomized trial is shown in Fig. 1.

### Statistical analysis

Data were expressed as the mean ± SD or mean ± SEM (for repeated-measures analysis). All statistical analyses were performed with statistical software SPSS, version 15.0 (Chicago, IL). For each variable, a repeated-measures analysis of variance was performed to identify subject-by-time profiles. A paired *t*-test was utilized to compare the clinical effect of UC-RBCs. Differences are considered to be significant with a P-value of less than 0.05.

## Results

### Effect of different pH levels and holding times

**Fig 2:**
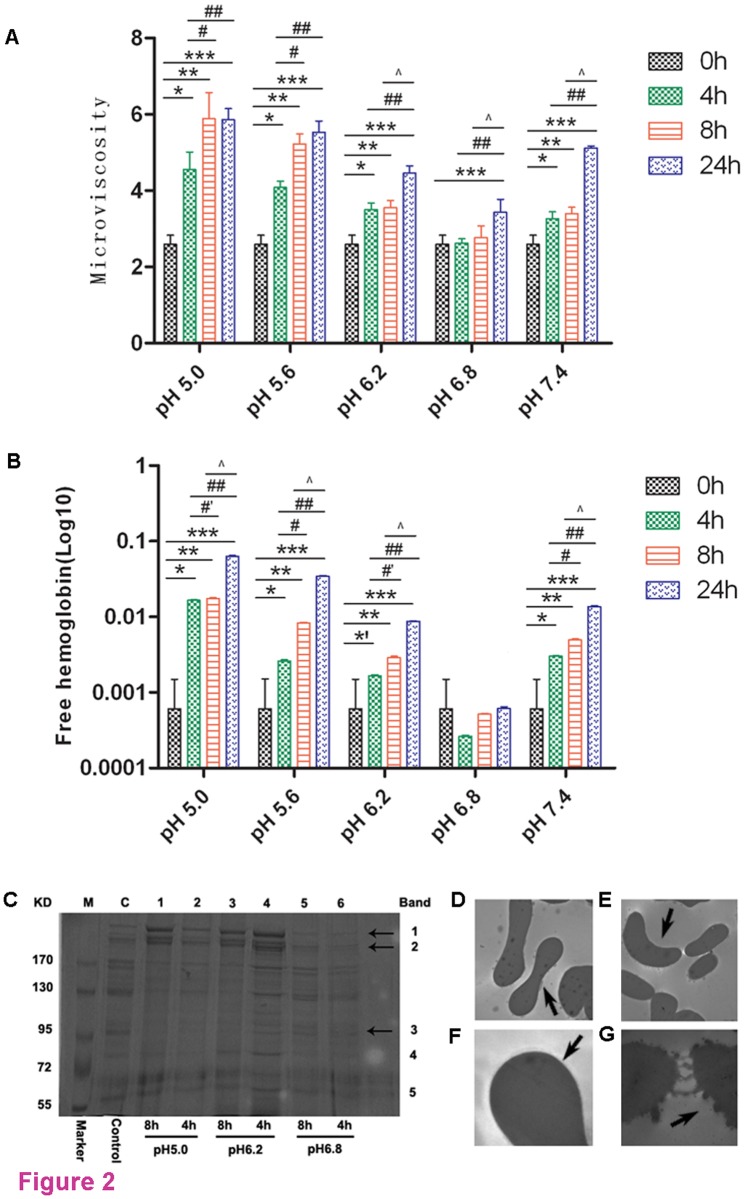
Effect of suspension pH and storage time on erythrocyte. **A. Effect of the suspension of pH and storage time on microviscosity**. *, p < 0.001, 0 h vs. 4 h; **, p < 0.001, 0 h vs. 8 h; ***, p < 0.001, 0 h vs. 24 h; #, p < 0.001, 4 h vs. 8 h; ##, p < 0.001, 4 h vs. 24 h; ^, p < 0.001, 8 h vs. 24 h. **B. Effect of the suspension of pH and storage time on free hemoglobin**. *, p < 0.001, *′, p < 0.05, 0 h vs. 4 h; **, p < 0.001, 0 h vs. 8 h; ***, p < 0.001, 0 h vs. 24 h; #, p < 0.001, #′, p < 0.05, 4 h vs. 8 h; ##, p < 0.001, 4 h vs. 24 h; ^, p < 0.001, 8 h vs. 24 h. **C. Analysis of erythrocyte membrane protein expression by SDS-PAGE electrophoresis**. Proteins were resolved by SDS-PAGE. Normal erythrocytes of healthy donors were used as the control. Lane 1 and lane 2 were the experimental group treated for 4 h or 8 h in an extracellular pH 5.0 environment; lane 3 and lane 4 were the experimental group treated for 4 h or 8 h in an extracellular pH 6.2 environment; lane 5 and lane 6 were the experimental group treated for 4 h or 8 h in an extracellular pH 6.8 environment. **D-G. Effects of suspension pH and storage time on erythrocyte membrane morphology**. D: normal biconcave red cells (20,000 x); E: Red cells that were stored for 4 h in ACD-B anticoagulant (pH 6.2) became invaginated and rough (20,000 x); F: red cells stored for 4 h in ACD-B anticoagulant of pH 6.8 were similar in morphology to normal erythrocytes (60,000 x); G: membrane dissolution of two spherical cells after storage for 24 h in ACD-B anticoagulant (pH 5.6; 60,000 x).

The results of this study indicated that the microviscosity (*η*) of erythrocyte membranes and the supernatant Hb levels increased in accordance with increased holding times (Fig. 2-A). They also showed that extracellular pH levels were lower, and the difference of the two indexes was larger. Among the 0 h vs. 4 h and 0 h vs. 8 h of the time-treated group, there was a significant difference in most pH-treated groups (p < 0.001), except in the pH 6.8 group. A significant difference in microviscosity was observed in all 0 h vs. 24 h and 4 h vs. 24 h groups (p < 0.001); while in the 4 h vs. 8 h group, only the pH 5.0 and pH 5.6 groups were significant (p < 0.001). By contrast, there was a significant difference in the 8 h vs. 24 h group, except for the pH 5.0 and pH 5.6 groups (p < 0.001).

With the exception of the pH 6.8 group, the concentration of free hemoglobin (FHb) showed an overall upward tendency during storage, with the 24 h of the treated groups higher than that in the 0 h untreated group, Among the 0 h vs. 4 h, 0 h vs. 8 h, 0 h vs. 24 h, 4 h vs. 8 h, 4 h vs. 24 h and 8 h vs. 24 h groups, the quantity of FHb was significant (p < 0.05). Changes were not seen in the pH 6.8 group at all time points (p > 0.05) (Fig. 2-B).

SDS-PAGE analysis revealed the effects of suspension pH and holding time on the erythrocyte membrane protein. We found that an aggregation of spectrin (bands 1 and 2) at low pH levels and large amounts of band 3 proteins were lost from the ghosts during treatment incubations. The oxidization and solubilization of band 3 protein increased with higher holding times and decreases in extracellular pH levels. The gels indicated no significant loss of band 3 protein between 4 h and 8 h of holding time in both the pH 6.8 and control groups (Fig. 2-C).

RBC morphology changes as a result of suspension pH and holding time were observed under the microscope. Along with increasing holding times and decreasing extracellular pH levels, the normal structure of red blood cells disappeared, RBCs gradually transformed their shape from normal smooth discocytes into stomatocytes, and even became spherical, with a large number of pseudopodia, debris and impurities appearing. This phenomenon arose when the 4 h group was stored in pH 6.2 anticoagulant, and the effect was enhanced with greater incubation times and lower extracellular pH values (Fig. 2-D-G).

### Component data

The UC-WB of 34 donors with vasovagal syncope or that had technical difficulties during venipuncture were used in a random order to prepare MAP UC-RBCs within 4 h, 8 h and 24 h of collection from volunteer donors attending the Jiangsu Province Blood Center (Nanjing, Jiangsu, China) or Yangzhou Blood Bank (Yangzhou, Jiangsu, China). All UC-RBCs units met the Chinese specification for Hct, Hb content and hemolysis. The osmotic fragility, pH, supernatant Na^+^ and 2,3-DPG showed a decreasing trend, while supernatant K^+^, EMP and hemolysis all had an upward trend. In short, pH changes from the baseline at each time point of the experimental groups were not significantly different. During storage, the supernatant K^+^ level increased from 4.34 ± 0.71 to 12.99 ± 4.23 in the 4 h group, whose potassium leakage was the smallest among the four groups and had a significant difference from 14 days in comparison with the 8 h and 24 h groups. Similar decreases that were not statistically different in sodium and 2,3-DPG levels were displayed throughout the storage period in all groups, but 2,3-DPG concentration decreased rapidly during the first 14 days. Although at the end of 35 days of storage, the percentage of hemolysis met regulatory requirements in all groups, there was a significant difference in the pairwise comparisons between the 4 h and 8 h groups, as well as the 4 h and 24 h groups from 14 days, and a significant difference in EMP was observed at 28 days between the 0 h and 4 h groups.

**Fig 3:**
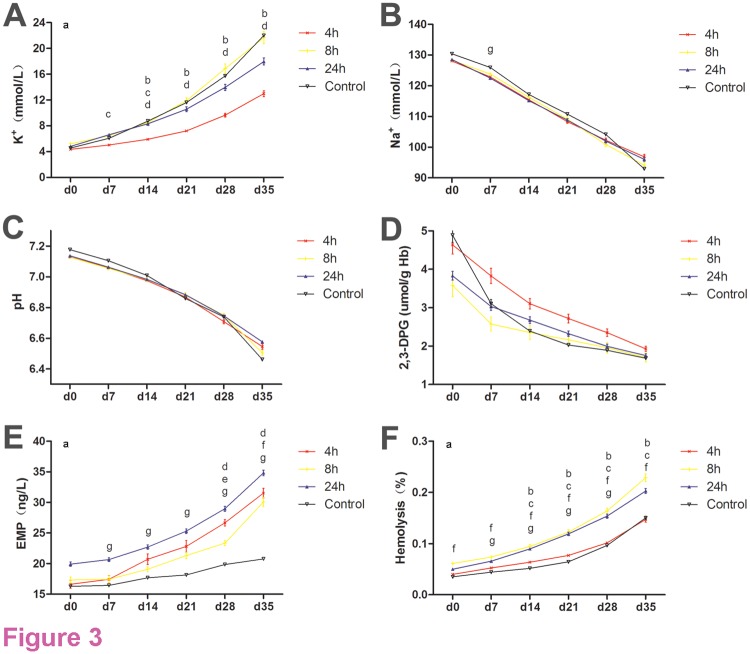
Major characteristics of UC-RBCs in vitro. **A**. Changes in K^+^ concentration over time during storage at 4°C; **B**. Changes in Na^+^ concentration over time during storage at 4°C; **C**. Changes in pH over time during storage at 4°C; **D**. Changes in 2,3-DPG over time during storage at 4°C; **E**. Changes in EMP over time during storage at 4°C; **F**. Changes in the percent of hemolysis over time during storage at 4°C. a: p < 0.05, comparison of between-subjects effects; b: p < 0.05, pairwise comparison at the same time point between 4 h and 8 h group; c: p < 0.05, pairwise comparison at the same time point between 4 h and 24 h group; d: p < 0.05, pairwise comparison at the same time point between 4 h and control group; e: p < 0.05, pairwise comparison at the same time point between 8 h and 24 h group; f: p < 0.05, pairwise comparison at the same time point between 8 h and control group; g: p < 0.05, pairwise comparison at the same time point between the 24 h and control groups.

The results of pH, supernatant K^+^, Na^+^, percentage hemolysis, EMP and 2,3-DPG are shown in Fig. 3. These findings demonstrated that, during 5 weeks of storage, the differences in the between-subjects effects of K^+^, free Hb and EMP were statistically significant (p-values = 0.041, 0.007 and 0.002, respectively); during 2 weeks of storage, most physical and chemical indicators of experimental groups did not show a statistical difference compared to controls; after 3 weeks of storage, the greatest difference was between the control group and the 24 h group for K^+^, FHb and free EMP, whereas the differences between the 4 h group and the comparable controls were less significant.

### Clinical assessment of UC-RBCs

**Fig 4:**
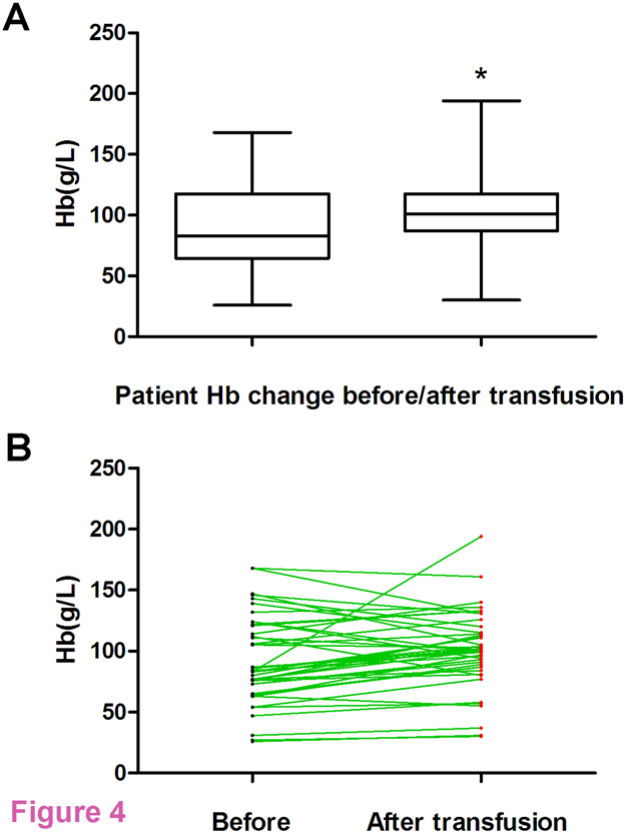
Change in Hb levels of patients before/after transfusion. **A**. Average Hb levels of 49 patients before and 24 h after transfusion. **B**. Diagram of Hb levels of 49 patients before and after transfusion. *: p < 0.0001, compared with the ‘before transfusion’ group.

A total of seven patients were excluded as they did not meet the study inclusion criteria; the clinical data of 49 patients were therefore studied in this trial. *Their diseases included* coronary heart disease, chronic nephropathy, iron-deficiency anemia, liver cirrhosis, femoral head necrosis, intussusception and malignancies, such as acute myeloid leukemia, gastric cancer, prostatic carcinoma, rectal cancer, uterine cervix cancer and colorectal cancer. The clinical data were shown in Table 1. There were 19 men (38.8%) and 30 women (61.2%), with a mean age of 53.71 ± 11.11 years (range: 23–65 years). The mean Hb count of patients before transfusion rose from 91.18 ± 34.84 g/L to 101.10 ± 32.95 g/L at 24 h after transfusion (t = 2.946, *p* < 0.0001), and clinical symptoms of hypoxia regressed or disappeared in all affected patients (Fig. 4). Clinical data from these 49 patients showed that the transfusion of UC-RBCs was satisfactory and no adverse reactions occurred either during transfusion of within 180 days thereafter.

**Table 1:** The clinical data of 49 patients transfusions with UC-RBCs.

Patient Number	Collection volume(ml)	Blood group	Holding time(h)	UC-RBC(U)*	Age of patient(Y)	Gender	Diagnosis	RBC transfused(U)	Hb of patient Before/After Transfusion(g/L)	RBC of patient Before/After Transfusion(10^12/L)	HCT of patient Before/After Transfusion	MCV of patient Before/After Transfusion(fl)	MCH of patient Before/After Transfusion(Pg)	MCHC of patient Before/After Transfusion(g/l)	Storage duration(d)	Adverse reactions
1	226	O	3.6	1	53	F	gastric cancer	5	47/58	2.20/1.90	0.22/0.18	101.0/94.0	31.0/31.0	314/329	2	N
2	324	B	3.4	1.5	65	F	hemorrhagic shock	3	27/30	3.10/3.21	0.27/0.28	85.0/87.0	30.0/31.0	355/355	6	N
3	235	AB	2.6	1	50	F	brain contusion	4	112/80	2.40/3.00	0.20/0.25	84.0/82.0	29.0/30.0	355/362	4	N
4	217	O	3.2	1	58	M	abdominal tumour	5	132/136	4.18/4.19	0.40/0.41	94.5/97.8	31.6/32.4	334/331	6	N
5	341	A	3.8	1.5	59	F	hypersplenism	63	83/111	2.40/3.80	0.20/0.32	84.0/84.0	29.0/29.0	349/343	5	N
6	328	A	3.7	1.5	53	M	coronary heart disease	9	106/126	3.60/4.27	0.32/0.38	88.5/88.1	29.4/29.5	334/333	6	N
7	219	A	2.2	1	26	F	gallbladder stone with chronic cholecystitis	5	47/58	4.79/3.30	0.44/0.27	92.6/83.0	31.4/29.0	339/359	7	N
8	236	A	2.9	1	40	F	fracture	11	105/104	4.20/2.90	0.35/0.24	83.3/80.7	26.7/28.3	320/350	4	N
9	228	A	1.9	1	48	F	cirrhosis	13	132/136	2.02/2.41	0.16/0.20	78.2/81.7	22.3/24.1	285/294	7	N
10	212	B	3.3	1	65	M	gastric cancer	4	54/57	2.00/3.10	0.17/0.26	87.0/86.0	28.0/29.0	326/339	3	N
11	245	B	3.8	1	61	M	rectal cancer	6.5	63/89	3.10/4.00	0.23/0.30	72.0/73.0	19.0/21.0	273/295	5	N
12	296	B	2.2	1.5	65	F	gastric cancer	28	73/93	2.60/3.10	0.22/0.26	86.0/86.0	28.0/29.0	335/339	5	N
13	237	B	3.2	1	34	M	primary hepatocarcinoma	2	146/131	4.83/4.07	0.43/0.39	89.4/95.5	30.2/32.4	338/339	7	N
14	215	A	2.8	1	55	F	rheumatic heart disease	18.5	114/140	4.04/4.70	0.36/0.43	89.6/91.0	28.2/29.0	315/324	6	N
15	254	O	3.8	1	59	M	coronary heart disease	5	147/105	4.75/3.40	0.43/0.29	89.5/84.0	30.9/30.0	346/359	5	N
16	231	O	2.7	1	53	F	necrosis of femoral head	6	121/133	4.39/3.20	0.42/0.29	95.7/88.0	31.9/31.0	333/351	4	N
17	289	B	3.1	1.5	65	F	rheumatic heart disease	3	80/112	2.90/3.76	0.26/0.34	89.0/89.6	27.0/29.8	305/332	4	N
18	328	B	3.6	1.5	59	M	acute non-lymphoid leukemia	6	77/84	2.75/2.96	0.23/0.24	81.8/80.4	28.0/26.0	342/345	7	N
19	293	O	3.3	1.5	61	M	intussusception	3	86/104	3.91/4.44	0.30/0.34	76.2/75.5	22.0/23.0	289/304	7	N
20	216	A	2.3	1	65	F	coronary heart disease	6	77/100	2.70/3.57	0.22/0.30	82.0/84.0	27.0/28.0	333/333	5	N
21	236	B	2.8	1	50	F	spinal disk herniation	3	143/120	4.72/3.71	0.42/0.34	89.6/92.0	30.3/32.5	338/352	2	N
22	218	A	1.8	1	62	M	prostate cancer	5	105/103	3.22/3.00	0.30/0.26	92.8/87.7	32.6/30.3	351/346	5	N
23	247	O	3.2	1	50	M	hemolytic anemia	4	31/37	3.41/4.42	0.25/0.34	73.1/76.7	22.6/22.6	309/295	1	N
24	287	B	3.5	1.5	23	F	iron-deficiency anemia	1.5	26/31	2.11/2.63	0.17/0.21	77.7/78.3	28.9/27.4	372/350	6	N
25	275	B	3.8	1.5	61	F	rheumatic heart disease	20	84/96	2.80/2.87	0.25/0.27	87.1/93.2	29.6/32.7	340/350	4	N
26	326	A	3.3	1.5	63	M	prostate hyperplasia	7.5	122/133	3.29/4.17	0.29/0.37	86.9/87.8	30.1/29.0	331/346	7	N
27	291	A	3.1	1.5	47	M	cerebellar space-occupying lesion	3	168/161	5.65/5.35	0.50/0.48	88.0/88.5	29.8/30.1	339/340	7	N
28	205	AB	3.2	1	60	F	brain stem tumor	3	87/99	2.94/3.43	0.27/0.31	90.5/88.9	29.6/28.9	327/325	1	N
29	209	B	3.9	1	51	F	uterine cervix cancer	8.5	83/111	4.44/4.73	0.41/0.41	91.9/87.3	30.0/28.3	326/324	3	N
30	223	O	3.8	1	53	F	multiple neurofibromatosis	32.5	63/97	1.86/4.05	0.19/0.39	104.3/96.3	33.3/31.1	320/323	5	N
31	283	A	3.4	1.5	60	F	gastric cancer	6	73/91	3.56/2.94	0.31/0.28	86.8/94.2	30.1/32.2	346/342	4	N
32	328	O	3.1	1.5	61	F	necrosis of femoral head	9	114/140	2.83/3.16	0.24/0.28	84.5/89.8	29.3/30.5	347/339	7	N
33	202	AB	2.8	1	65	F	chronic nephropathy	11.5	54/77	1.82/2.76	0.16/0.23	88.6/83.0	29.6/27.2	334/328	7	N
34	325	B	3.8	1.5	44	F	cerebellar space-occupying lesion	3	124/94	3.79/3.06	0.37/0.29	96.7/95.4	32.7/32.3	338/339	6	N
35	217	B	2.9	1	63	F	liver cirrhosis	2	64/87	2.79/3.58	0.19/0.27	69.5/74.9	22.9/24.3	330、325	4	N
36	214	AB	3.2	1	48	M	gastric cancer	15	86/102	3.55/2.28	0.36/0.19	100.1/85.1	35.1/30.7	350/361	6	N
37	294	A	3.1	1.5	23	F	intestinal fistula	3.5	168/131	4.18/4.12	0.36/0.34	84.9/83.6	27.2/31.0	321/370	7	N
38	228	B	3.3	1	47	M	glioma	2	87/99	3.32/3.90	0.27/0.32	81.3/81.3	27.1/26.4	333/324	7	N
39	294	A	3.7	1.5	58	F	duodenal carcinoma	3	63/97	3.45/3.74	0.31/0.34	89.3/90.6	30.1/30.5	338/336	6	N
40	316	O	3.9	1.5	65	F	rheumatic heart disease	1.5	80/112	2.90/2.99	0.23/0.24	78.0/80.9	27.6/27.0	353/333	7	N
41	284	O	3.6	1.5	61	F	cerebral trauma	5.5	77/104	2.67/2.97	0.23/0.26	85.0/86.9	28.9/29.0	340/334	3	N
42	289	AB	3.6	1.5	59	F	abdominal tumour	1.5	139/115	4.79/4.10	0.45/0.37	93.7/90.1	29.0/30.7	310/340	4	N
43	218	O	3.2	1	58	F	intestinal fistula	20	124/99	3.32/3.00	0.29/0.26	88.3/88	29.5/30.0	334/341	2	N
44	328	AB	3.1	1.5	60	M	acute myeloid leukemia	15	63/55	1.74/1.49	0.19/0.15	108.0/102.0	36.2/36.3	335/356	6	N
45	281	A	3.7	1.5	58	M	rib fracture	16.5	83/194	2.56/4.05	0.23/0.36	90.2/89.4	31.3/30.6	346/342	7	N
46	317	B	3.3	1.5	64	F	colon cancer	8	65/87	2.77/2.76	0.22/0.24	78.0/87.7	24.9/29.3	319/335	4	N
47	228	AB	3.7	1	32	M	pancreatitis	48	76/81	2.15/2.51	0.20/0.24	93.9/94.9	31.2/30.7	332/323	2	N
48	207	A	3.6	1	45	M	multiple fracture	18	76/113	2.87/1.77	0.26/0.18	92.0/98.6	30.7/32.2	333/327	4	N
49	238	O	3.2	1	47	M	intestinal pertbration	7	111/101	2.46/2.85	0.25/0.28	99.8/96.6	32.5/30.9	326/320	1	N

* 1 unit blood component is prepared with 200ml WB in China

## Discussion

A large number of investigations have shown that cell pH, a fundamental biological property that independently modulates erythrocyte membrane structure and function, is an important factor that affects red cell viability. It has been reported that the pH value of the extracellular medium affects erythrocyte morphology and functions by influencing its membrane [10–12]. There is also evidence to suggest that the initial pH in RBCs might have an effect throughout the entire storage period of RBCs [13, 14]. Low pH levels could cause an aggregation of integral membrane proteins [11] and alter the activity of certain enzymes and biochemical pathways [15]. The inorganic phosphate in RBC anticoagulant or additive solution may also reduce phosphate loss, induce the formation of organic phosphate esters together with an increase of pH of solution, and reduce the depletion of 2,3-DPG., Underweight units generally deviate from standard ratios of whole blood to anticoagulant, and their suspension pH is less than that of normal units. We measured the pH value of 55 under-collected WB units whose real collected volumes were greater than 200 mL: the mean values within 24 h after collection of these samples were 6.34 ± 0.26, and we therefore speculated that this imbalance leads to a more acidic environment in under-collected units that is a principal cause of damage to RBCs. Furthermore, as MAP contains sodium phosphate and has a higher pH value than ACD-B, we hypothesized that collecting low volume units should be more compatible with the ACD-B/MAP system.

To elucidate how the supernatant pH and holding time contribute to deleterious changes to RBCs, we have investigated membrane fluidity, proteins and RBC morphology by exposing erythrocytes to various pH values as well as holding times. Our study indicated that a loss of membrane fluidity and protein was associated with acidification of the suspension medium: the lower was the pH value, the greater was the decrease of membrane fluidity and membrane proteins in the acidic range of pH (5.0–6.8). Increased holding times, however, had the opposite effect. From Fig. 2, we can see that some changes in membrane proteins in different pH suspensions were found in the constitution of the major bands of the RBCs, such as bands 1, 2 and 3; the intensities of these bands changed markedly as the pH value deviated from the neutral one. The aggregation of integral membrane proteins and volume changes of functional proteins at low pH levels can affect the membrane fluidity, morphology and function of erythrocytes.

Although many investigations have demonstrated that the morphology of human erythrocytes changes in accordance with variations in the extracellular medium pH, the threshold value that had an effect on morphological changes was not identical. Gedde et al. consider that intact cells can maintain normal shape in a pH range of 6.3–7.9 [16, 17]; this pH range is wider than the 7.2–7.4 range reported by Wu et al. [18] that maintains the biconcave discoid shape of intact erythrocytes. Our investigation was not consistent with these above-mentioned results: we found that erythrocytes had no noticeable changes in morphology in the investigated pH range of 6.8–7.4; moreover, we found that some erythrocytes became spherical when incubated in an extracellular medium of pH 6.2 rather than pH 4.82 reported by Wu et al. In this study, the highest value of supernatant Hb was 65.9 ± 0.9 mg/L after incubation of 24 h in pH 5.0 medium; these result were similar to an earlier report by Arvinte et al. [19], which indicated that erythrocytes undergo moderate and slow hemolysis due to acidic intracellular environments. Therefore, we hypothesized that a suspension environment of UC-WB with a lower pH than that of normal whole blood might impair the quality of RBCs.

In general, for a unit to be usable, the current regulations require that the quantity collected be within 10% of the intended collection volume to ensure that prepackaged anticoagulants are diluted appropriately and that the expected standard dose for a unit of blood is achieved. A non-standard donation (either under- or overweight) should usually be removed from the supply or be labeled accordingly if released for clinical use. Given that there are three models of bags (200, 300 and 400 ml) for whole blood collection in China, many low volume units greater than 200 or 300 ml are discarded because of non-compliance with Chinese regulatory standards. For example, if a donor had planned to donate 400 mL whole blood and has even donated more than 300 mL of blood, but the amount of blood does not reach 360 mL, or a donor had planned to donate 300 mL whole blood and even has donated more than 200 mL of blood, but the amount of blood does not reach 270 mL, then the blood donated has to be described as an underweight unit. If extrapolated to the 2011 national annual blood collection of 2.082 x 10^7^ U, this represents a loss equivalent to the annual collection of a large provincial blood center. However, underweight units are allowed to be used in clinical transfusion in the USA and many European countries. For example, 300 to 404 mL of whole blood collected into an anticoagulant volume calculated for 450 ± 45 mL or 333 to 449 mL of whole blood collected into an anticoagulant volume calculated for 500 mL ± 50 mL is now eligible to be labeled and released as “red blood cells—low volume” by AABB [20]. This policy gave us the guidelines for determining the limit of UC-WB used to prepare UC-RBCs in our investigation: the units of UC-WB volume that were more than 66% of the intended collection, (i.e., real collected volume >198 ml in a 300 ml bag; >264 ml in a 400 ml bag), were selected. These were randomly divided into 4 h, 8 h and 24 h groups to prepare MAP UC-RBCs, as blood components in China are generally prepared from WB between 6 and 8 h after phlebotomy. To avoid damage to erythrocytes by the leukocyte depletion filter, no UC-RBCs were leukoreduced.

As the WB holding time before separation into components is a factor that influences the quality of red cells [21], we determined the major physical and chemical indicators of UC-RBCs to describe the effects of preparation time on the quality of MAP UC-RBC units. By analyzing the differences in data at the same time points across the four groups, we found that the difference between the 4 h group and the comparable controls was less significant among the three groups with between-group differences: there was no difference in the levels of hemolysis between the 4 h and control groups over the entire storage period and any index of the 4 h group. This was with the exception of K^+^ concentration, which did not differ from that of the control group during 14 days of storage, while the difference between the 24 h and control groups was significant for supernatant EMP from day 7 onwards. As the K^+^ concentration of the 4 h group was lower than the normal control group and it turned out that a significant difference existed between the 24 h and control groups for supernatant EMP and hemolysis from the 7^th^ storage day, we speculated that shortening the time between the collection and separation of RBC from the acidic suspension helps to reduce the leakage of K^+^ in RBCs, and a longer holding time or greater separation time was unfavorable to maintaining the structure and function of erythrocytes. These results will be helpful to provide a reliable basis for preparing and transfusing MAP UC-RBCs. We therefore undertook clinical experiments using blood components prepared within 4 h after collection and stored within 1 week among patients who had given informed consent. We found that all MAP UC-RBCs units were transfused without creating clinical confusion or patient risk, and satisfactory clinical outcomes were achieved that demonstrated the safety and effectiveness of this blood product.

## Conclusions

Although the sample size was relatively small, and the trial was conducted in three centers, we believe that the recovery of UC-WB would be a welcome addition to our limited blood resources. Our experimental and clinical data might serve as a reference for revising the actual guidelines or regulatory standards in order to improve the shortage of blood in our country. Therefore, we propose the following recommendations: 1) ACD-B anticoagulated UC-WB that is greater than 66% of the intended collection volume can be used to prepare the MAP UC-RBCs; 2) MAP UC-RBCs prepared within 4 h after collection and stored within 1 week can maintain the quality of standard RBC under the precondition of a shortened storage period; and 3) such RBCs are effective and safe for transfused patients and can be utilized in transfusion therapies.

## Supporting Information

S1 CONSORT Checklist Checklist of this paper. (DOC)

S1 Protocol Protocol of this trial. The protocol of experimental and clinical studing on WB/UC-WB RBCs is shown by flow chart.
(DOC)

S1 Table MAP UC-RBCs and RBCs component data. (XLS)

S1 File Multi-variate test of repetitive measure ANOVA. (DOC)

S2 File EHN Working Party on Definitions of ATEs. (PDF)
