# Supplementary material for: Erythrocyte Concentrates Recovered from Under-Collected Whole Blood: Experimental and Clinical Results
Source: PLoS One. 2015 Feb 23;10(2):e0117928. doi: 10.1371/journal.pone.0117928 (PMC4338272; doi:10.1371/journal.pone.0117928)
Supplement: S1 Protocol — The protocol of experimental and clinical studing on WB/UC-WB RBCs is shown by flow chart. (DOC) [file pone.0117928.s002.doc]

**Study protocol**

**WB**

**UC-WB**

>66% volume of the labeled collection

holding times： 4, 8 and 24 h

**WB / UC-WB RBCs**

Evaluation of the safety and efficacy

holding times≤4h，stored times≤1w

**Clinical trial**

**In vitro study**

0, 7, 14, 21, 28 and 35d

**Chemical and physical index**

(RBC Count, Hct, MCV, K+, Na+, Cl-, FHb, 2.3-DPG and supernatant EMP)

**Indicators of structure and function**

(osmotic fragility, morphology, membrane fluidity, membrane molecules)

Change in the patient’s Hb before and 24 h after transfusion

Adverse reactions during and within 180 days of transfusion
