# Supplementary material for: Erythrocyte Concentrates Recovered from Under-Collected Whole Blood: Experimental and Clinical Results
Source: PLoS One. 2015 Feb 23;10(2):e0117928. doi: 10.1371/journal.pone.0117928 (PMC4338272; doi:10.1371/journal.pone.0117928)
Supplement: S1 File — (DOC) [file pone.0117928.s004.doc]

**Multi-variate test of repetitive measure ANOVA:**

**1. K^+^:**

**A.Pairwise Comparisons between groups:**

| (I) Group | (J) Group | Mean Difference (I-J) | Std. Error | Sig.(a) | 95% Confidence Interval for Difference(a) | |
| --- | --- | --- | --- | --- | --- | --- |
|  |  |  |  |  | Upper Bound | Lower Bound |
| 1 | 2 | -4.255 | 1.553 | .055 | -8.571 | .061 |
|  | 3 | -2.853 | 1.425 | .314 | -6.813 | 1.108 |
|  | 4 | -3.929 | 1.593 | .109 | -8.357 | .499 |
| 2 | 1 | 4.255 | 1.553 | .055 | -.061 | 8.571 |
|  | 3 | 1.402 | 1.380 | 1.000 | -2.433 | 5.237 |
|  | 4 | .325 | 1.553 | 1.000 | -3.990 | 4.641 |
| 3 | 1 | 2.853 | 1.425 | .314 | -1.108 | 6.813 |
|  | 2 | -1.402 | 1.380 | 1.000 | -5.237 | 2.433 |
|  | 4 | -1.077 | 1.425 | 1.000 | -5.037 | 2.884 |
| 4 | 1 | 3.929 | 1.593 | .109 | -.499 | 8.357 |
|  | 2 | -.325 | 1.553 | 1.000 | -4.641 | 3.990 |
|  | 3 | 1.077 | 1.425 | 1.000 | -2.884 | 5.037 |

Based on estimated marginal means

a Adjustment for multiple comparisons: Bonferroni.

**B. Multiple Comparisons at the same time point among groups:**

| Dependent Variable |  | (I) Group | (J) Group | Mean Difference (I-J) | Std. Error | Sig. | 95% Confidence Interval | |
| --- | --- | --- | --- | --- | --- | --- | --- | --- |
|  |  |  |  | Lower Bound | Upper Bound | Lower Bound | Upper Bound | Lower Bound |
| d0 | LSD | 1 | 2 | -.8656 | .33020 | .075 | -1.7834 | .0523 |
|  |  |  | 3 | -.4622 | .30302 | .135 | -1.0751 | .1507 |
|  |  |  | 4 | -.2000 | .33878 | .558 | -.8853 | .4853 |
|  |  | 2 | 1 | .8656 | .33020 | .075 | -.0523 | 1.7834 |
|  |  |  | 3 | .4033 | .29339 | .177 | -.1901 | .9968 |
|  |  |  | 4 | .6656 | .33020 | .051 | -.0023 | 1.3335 |
|  |  | 3 | 1 | .4622 | .30302 | .135 | -.1507 | 1.0751 |
|  |  |  | 2 | -.4033 | .29339 | .177 | -.9968 | .1901 |
|  |  |  | 4 | .2622 | .30302 | .392 | -.3507 | .8751 |
|  |  | 4 | 1 | .2000 | .33878 | .558 | -.4853 | .8853 |
|  |  |  | 2 | -.6656 | .33020 | .051 | -1.3335 | .0023 |
|  |  |  | 3 | -.2622 | .30302 | .392 | -.8751 | .3507 |
| d7 | LSD | 1 | 2 | -1.4478 | .74080 | .058 | -2.9462 | .0506 |
|  |  |  | 3 | -1.5978(*) | .67980 | .024 | -2.9728 | -.2227 |
|  |  |  | 4 | -1.0444 | .76004 | .177 | -2.5818 | .4929 |
|  |  | 2 | 1 | 1.4478 | .74080 | .058 | -.0506 | 2.9462 |
|  |  |  | 3 | -.1500 | .65822 | .821 | -1.4814 | 1.1814 |
|  |  |  | 4 | .4033 | .74080 | .589 | -1.0951 | 1.9017 |
|  |  | 3 | 1 | 1.5978(*) | .67980 | .024 | .2227 | 2.9728 |
|  |  |  | 2 | .1500 | .65822 | .821 | -1.1814 | 1.4814 |
|  |  |  | 4 | .5533 | .67980 | .421 | -.8217 | 1.9284 |
|  |  | 4 | 1 | 1.0444 | .76004 | .177 | -.4929 | 2.5818 |
|  |  |  | 2 | -.4033 | .74080 | .589 | -1.9017 | 1.0951 |
|  |  |  | 3 | -.5533 | .67980 | .421 | -1.9284 | .8217 |
|  |  |  | 3 | -.5533 | .66169 | .960 | -2.5074 | 1.4008 |
| d14 | LSD | 1 | 2 | -2.6100(*) | 1.12529 | .026 | -4.8861 | -.3339 |
|  |  |  | 3 | -2.4000(*) | 1.03264 | .025 | -4.4887 | -.3113 |
|  |  |  | 4 | -2.8333(*) | 1.15453 | .019 | -5.1686 | -.4981 |
|  |  | 2 | 1 | 2.6100(*) | 1.12529 | .026 | .3339 | 4.8861 |
|  |  |  | 3 | .2100 | .99985 | .835 | -1.8124 | 2.2324 |
|  |  |  | 4 | -.2233 | 1.12529 | .844 | -2.4995 | 2.0528 |
|  |  | 3 | 1 | 2.4000(*) | 1.03264 | .025 | .3113 | 4.4887 |
|  |  |  | 2 | -.2100 | .99985 | .835 | -2.2324 | 1.8124 |
|  |  |  | 4 | -.4333 | 1.03264 | .677 | -2.5220 | 1.6554 |
|  |  | 4 | 1 | 2.8333(*) | 1.15453 | .019 | .4981 | 5.1686 |
|  |  |  | 2 | .2233 | 1.12529 | .844 | -2.0528 | 2.4995 |
|  |  |  | 3 | .4333 | 1.03264 | .677 | -1.6554 | 2.5220 |
|  |  |  | 3 | .4333 | .95899 | .998 | -2.4161 | 3.2828 |
| d21 | LSD | 1 | 2 | -4.6989(*) | 1.82083 | .014 | -8.3819 | -1.0159 |
|  |  |  | 3 | -3.3489 | 1.67091 | .052 | -6.7286 | .0308 |
|  |  |  | 4 | -4.4000(*) | 1.86814 | .024 | -8.1787 | -.6213 |
|  |  | 2 | 1 | 4.6989(*) | 1.82083 | .014 | 1.0159 | 8.3819 |
|  |  |  | 3 | 1.3500 | 1.61785 | .409 | -1.9224 | 4.6224 |
|  |  |  | 4 | .2989 | 1.82083 | .870 | -3.3841 | 3.9819 |
|  |  | 3 | 1 | 3.3489 | 1.67091 | .052 | -.0308 | 6.7286 |
|  |  |  | 2 | -1.3500 | 1.61785 | .409 | -4.6224 | 1.9224 |
|  |  |  | 4 | -1.0511 | 1.67091 | .533 | -4.4308 | 2.3286 |
|  |  | 4 | 1 | 4.4000(*) | 1.86814 | .024 | .6213 | 8.1787 |
|  |  |  | 2 | -.2989 | 1.82083 | .870 | -3.9819 | 3.3841 |
|  |  |  | 3 | 1.0511 | 1.67091 | .533 | -2.3286 | 4.4308 |
| d28 | LSD | 1 | 2 | -7.2256(*) | 2.58651 | .008 | -12.4573 | -1.9939 |
|  |  |  | 3 | -4.3156 | 2.37354 | .077 | -9.1165 | .4854 |
|  |  |  | 4 | -6.0878(*) | 2.65370 | .027 | -11.4554 | -.7202 |
|  |  | 2 | 1 | 7.2256(*) | 2.58651 | .008 | 1.9939 | 12.4573 |
|  |  |  | 3 | 2.9100 | 2.29817 | .213 | -1.7385 | 7.5585 |
|  |  |  | 4 | 1.1378 | 2.58651 | .662 | -4.0939 | 6.3695 |
|  |  | 3 | 1 | 4.3156 | 2.37354 | .077 | -.4854 | 9.1165 |
|  |  |  | 2 | -2.9100 | 2.29817 | .213 | -7.5585 | 1.7385 |
|  |  |  | 4 | -1.7722 | 2.37354 | .460 | -6.5732 | 3.0287 |
|  |  | 4 | 1 | 6.0878(*) | 2.65370 | .027 | .7202 | 11.4554 |
|  |  |  | 2 | -1.1378 | 2.58651 | .662 | -6.3695 | 4.0939 |
|  |  |  | 3 | 1.7722 | 2.37354 | .460 | -3.0287 | 6.5732 |
| d35 | LSD | 1 | 2 | -8.6811(*) | 3.21629 | .010 | -15.1867 | -2.1756 |
|  |  |  | 3 | -4.9911 | 2.95147 | .099 | -10.9610 | .9788 |
|  |  |  | 4 | -9.0111(*) | 3.29984 | .009 | -15.6857 | -2.3365 |
|  |  | 2 | 1 | 8.6811(*) | 3.21629 | .010 | 2.1756 | 15.1867 |
|  |  |  | 3 | 3.6900 | 2.85775 | .204 | -2.0903 | 9.4703 |
|  |  |  | 4 | -.3300 | 3.21629 | .919 | -6.8356 | 6.1756 |
|  |  | 3 | 1 | 4.9911 | 2.95147 | .099 | -.9788 | 10.9610 |
|  |  |  | 2 | -3.6900 | 2.85775 | .204 | -9.4703 | 2.0903 |
|  |  |  | 4 | -4.0200 | 2.95147 | .181 | -9.9899 | 1.9499 |
|  |  | 4 | 1 | 9.0111(*) | 3.29984 | .009 | 2.3365 | 15.6857 |
|  |  |  | 2 | .3300 | 3.21629 | .919 | -6.1756 | 6.8356 |
|  |  |  | 3 | 4.0200 | 2.95147 | .181 | -1.9499 | 9.9899 |

Based on observed means.

* The mean difference is significant at the .05 level.

**2. Na^+^:**

**A.Pairwise Comparisons between groups:**

| (I) Group | (J) Group | Mean Difference (I-J) | Std. Error | Sig.(a) | 95% Confidence Interval for Difference(a) | |
| --- | --- | --- | --- | --- | --- | --- |
|  |  |  |  |  | Upper Bound | Lower Bound |
| 1 | 2 | .194 | 1.926 | 1.000 | -5.161 | 5.548 |
|  | 3 | .136 | 1.768 | 1.000 | -4.777 | 5.050 |
|  | 4 | -1.269 | 1.976 | 1.000 | -6.763 | 4.224 |
| 2 | 1 | -.194 | 1.926 | 1.000 | -5.548 | 5.161 |
|  | 3 | -.057 | 1.712 | 1.000 | -4.815 | 4.700 |
|  | 4 | -1.463 | 1.926 | 1.000 | -6.817 | 3.892 |
| 3 | 1 | -.136 | 1.768 | 1.000 | -5.050 | 4.777 |
|  | 2 | .057 | 1.712 | 1.000 | -4.700 | 4.815 |
|  | 4 | -1.405 | 1.768 | 1.000 | -6.319 | 3.508 |
| 4 | 1 | 1.269 | 1.976 | 1.000 | -4.224 | 6.763 |
|  | 2 | 1.463 | 1.926 | 1.000 | -3.892 | 6.817 |
|  | 3 | 1.405 | 1.768 | 1.000 | -3.508 | 6.319 |

Based on estimated marginal means

a Adjustment for multiple comparisons: Bonferroni.

**B. Multiple Comparisons at the same time point among groups:**

| Dependent Variable |  | (I) Group | (J) Group | Mean Difference (I-J) | Std. Error | Sig. | 95% Confidence Interval | |
| --- | --- | --- | --- | --- | --- | --- | --- | --- |
|  |  |  |  |  |  |  | Upper Bound | Lower Bound |
| d0 | LSD | 1 | 2 | -.2689 | 1.42678 | .851 | -3.1548 | 2.6170 |
|  |  |  | 3 | -.4956 | 1.30930 | .707 | -3.1439 | 2.1528 |
|  |  |  | 4 | -2.3667 | 1.46385 | .114 | -5.3276 | .5942 |
|  |  | 2 | 1 | .2689 | 1.42678 | .851 | -2.6170 | 3.1548 |
|  |  |  | 3 | -.2267 | 1.26773 | .859 | -2.7909 | 2.3376 |
|  |  |  | 4 | -2.0978 | 1.42678 | .150 | -4.9837 | .7882 |
|  |  | 3 | 1 | .4956 | 1.30930 | .707 | -2.1528 | 3.1439 |
|  |  |  | 2 | .2267 | 1.26773 | .859 | -2.3376 | 2.7909 |
|  |  |  | 4 | -1.8711 | 1.30930 | .161 | -4.5194 | .7772 |
|  |  | 4 | 1 | 2.3667 | 1.46385 | .114 | -.5942 | 5.3276 |
|  |  |  | 2 | 2.0978 | 1.42678 | .150 | -.7882 | 4.9837 |
|  |  |  | 3 | 1.8711 | 1.30930 | .161 | -.7772 | 4.5194 |
| d7 | LSD | 1 | 2 | -.9756 | 1.62115 | .551 | -4.2546 | 2.3035 |
|  |  |  | 3 | .3778 | 1.48767 | .801 | -2.6313 | 3.3869 |
|  |  |  | 4 | -3.1000 | 1.66327 | .070 | -6.4643 | .2643 |
|  |  | 2 | 1 | .9756 | 1.62115 | .551 | -2.3035 | 4.2546 |
|  |  |  | 3 | 1.3533 | 1.44043 | .353 | -1.5602 | 4.2669 |
|  |  |  | 4 | -2.1244 | 1.62115 | .198 | -5.4035 | 1.1546 |
|  |  | 3 | 1 | -.3778 | 1.48767 | .801 | -3.3869 | 2.6313 |
|  |  |  | 2 | -1.3533 | 1.44043 | .353 | -4.2669 | 1.5602 |
|  |  |  | 4 | -3.4778(*) | 1.48767 | .025 | -6.4869 | -.4687 |
|  |  | 4 | 1 | 3.1000 | 1.66327 | .070 | -.2643 | 6.4643 |
|  |  |  | 2 | 2.1244 | 1.62115 | .198 | -1.1546 | 5.4035 |
|  |  |  | 3 | 3.4778(*) | 1.48767 | .025 | .4687 | 6.4869 |
|  |  |  | 3 | 3.4778 | 1.23242 | .067 | -.1748 | 7.1304 |
| d14 | LSD | 1 | 2 | -.8356 | 1.96533 | .673 | -4.8108 | 3.1397 |
|  |  |  | 3 | .3111 | 1.80351 | .864 | -3.3368 | 3.9591 |
|  |  |  | 4 | -1.7222 | 2.01639 | .398 | -5.8008 | 2.3563 |
|  |  | 2 | 1 | .8356 | 1.96533 | .673 | -3.1397 | 4.8108 |
|  |  |  | 3 | 1.1467 | 1.74624 | .515 | -2.3854 | 4.6788 |
|  |  |  | 4 | -.8867 | 1.96533 | .654 | -4.8619 | 3.0886 |
|  |  | 3 | 1 | -.3111 | 1.80351 | .864 | -3.9591 | 3.3368 |
|  |  |  | 2 | -1.1467 | 1.74624 | .515 | -4.6788 | 2.3854 |
|  |  |  | 4 | -2.0333 | 1.80351 | .266 | -5.6813 | 1.6146 |
|  |  | 4 | 1 | 1.7222 | 2.01639 | .398 | -2.3563 | 5.8008 |
|  |  |  | 2 | .8867 | 1.96533 | .654 | -3.0886 | 4.8619 |
|  |  |  | 3 | 2.0333 | 1.80351 | .266 | -1.6146 | 5.6813 |
| d21 | LSD | 1 | 2 | -.8400 | 2.68373 | .756 | -6.2684 | 4.5884 |
|  |  |  | 3 | -.5200 | 2.46276 | .834 | -5.5014 | 4.4614 |
|  |  |  | 4 | -2.4556 | 2.75345 | .378 | -8.0249 | 3.1138 |
|  |  | 2 | 1 | .8400 | 2.68373 | .756 | -4.5884 | 6.2684 |
|  |  |  | 3 | .3200 | 2.38456 | .894 | -4.5032 | 5.1432 |
|  |  |  | 4 | -1.6156 | 2.68373 | .551 | -7.0439 | 3.8128 |
|  |  | 3 | 1 | .5200 | 2.46276 | .834 | -4.4614 | 5.5014 |
|  |  |  | 2 | -.3200 | 2.38456 | .894 | -5.1432 | 4.5032 |
|  |  |  | 4 | -1.9356 | 2.46276 | .437 | -6.9170 | 3.0458 |
|  |  | 4 | 1 | 2.4556 | 2.75345 | .378 | -3.1138 | 8.0249 |
|  |  |  | 2 | 1.6156 | 2.68373 | .551 | -3.8128 | 7.0439 |
|  |  |  | 3 | 1.9356 | 2.46276 | .437 | -3.0458 | 6.9170 |
| d28 | LSD | 1 | 2 | 1.4078 | 3.10712 | .653 | -4.8770 | 7.6925 |
|  |  |  | 3 | .3178 | 2.85129 | .912 | -5.4495 | 6.0851 |
|  |  |  | 4 | -1.8722 | 3.18784 | .560 | -8.3202 | 4.5758 |
|  |  | 2 | 1 | -1.4078 | 3.10712 | .653 | -7.6925 | 4.8770 |
|  |  |  | 3 | -1.0900 | 2.76075 | .695 | -6.6741 | 4.4941 |
|  |  |  | 4 | -3.2800 | 3.10712 | .298 | -9.5647 | 3.0047 |
|  |  | 3 | 1 | -.3178 | 2.85129 | .912 | -6.0851 | 5.4495 |
|  |  |  | 2 | 1.0900 | 2.76075 | .695 | -4.4941 | 6.6741 |
|  |  |  | 4 | -2.1900 | 2.85129 | .447 | -7.9573 | 3.5773 |
|  |  | 4 | 1 | 1.8722 | 3.18784 | .560 | -4.5758 | 8.3202 |
|  |  |  | 2 | 3.2800 | 3.10712 | .298 | -3.0047 | 9.5647 |
|  |  |  | 3 | 2.1900 | 2.85129 | .447 | -3.5773 | 7.9573 |
| d35 | LSD | 1 | 2 | 2.6733 | 3.42265 | .439 | -4.2496 | 9.5963 |
|  |  |  | 3 | .8267 | 3.14083 | .794 | -5.5263 | 7.1796 |
|  |  |  | 4 | 3.9022 | 3.51156 | .273 | -3.2006 | 11.0050 |
|  |  | 2 | 1 | -2.6733 | 3.42265 | .439 | -9.5963 | 4.2496 |
|  |  |  | 3 | -1.8467 | 3.04110 | .547 | -7.9979 | 4.3045 |
|  |  |  | 4 | 1.2289 | 3.42265 | .721 | -5.6941 | 8.1518 |
|  |  | 3 | 1 | -.8267 | 3.14083 | .794 | -7.1796 | 5.5263 |
|  |  |  | 2 | 1.8467 | 3.04110 | .547 | -4.3045 | 7.9979 |
|  |  |  | 4 | 3.0756 | 3.14083 | .334 | -3.2774 | 9.4285 |
|  |  | 4 | 1 | -3.9022 | 3.51156 | .273 | -11.0050 | 3.2006 |
|  |  |  | 2 | -1.2289 | 3.42265 | .721 | -8.1518 | 5.6941 |
|  |  |  | 3 | -3.0756 | 3.14083 | .334 | -9.4285 | 3.2774 |

Based on observed means.

* The mean difference is significant at the .05 level.

**3.pH**

**A.Pairwise Comparisons between groups:**

| (I) Group | (J) Group | Mean Difference (I-J) | Std. Error | Sig.(a) | 95% Confidence Interval for Difference(a) | |
| --- | --- | --- | --- | --- | --- | --- |
|  |  |  |  |  | Upper Bound | Lower Bound |
| 1 | 2 | -.003 | .040 | 1.000 | -.114 | .109 |
|  | 3 | -.017 | .037 | 1.000 | -.120 | .085 |
|  | 4 | -.011 | .041 | 1.000 | -.125 | .103 |
| 2 | 1 | .003 | .040 | 1.000 | -.109 | .114 |
|  | 3 | -.014 | .036 | 1.000 | -.113 | .085 |
|  | 4 | -.008 | .040 | 1.000 | -.119 | .104 |
| 3 | 1 | .017 | .037 | 1.000 | -.085 | .120 |
|  | 2 | .014 | .036 | 1.000 | -.085 | .113 |
|  | 4 | .006 | .037 | 1.000 | -.096 | .109 |
| 4 | 1 | .011 | .041 | 1.000 | -.103 | .125 |
|  | 2 | .008 | .040 | 1.000 | -.104 | .119 |
|  | 3 | -.006 | .037 | 1.000 | -.109 | .096 |

Based on estimated marginal means

a Adjustment for multiple comparisons: Bonferroni.

B. **Multiple Comparisons**

| Dependent Variable |  | (I) Group | (J) Group | Mean Difference (I-J) | Std. Error | Sig. | 95% Confidence Interval | |
| --- | --- | --- | --- | --- | --- | --- | --- | --- |
|  |  |  |  |  |  |  | Upper Bound | Lower Bound |
| d0 | LSD | 1 | 2 | .0019 | .03110 | .952 | -.0610 | .0648 |
|  |  |  | 3 | -.0078 | .02854 | .787 | -.0655 | .0499 |
|  |  |  | 4 | -.0478 | .03190 | .142 | -.1123 | .0168 |
|  |  | 2 | 1 | -.0019 | .03110 | .952 | -.0648 | .0610 |
|  |  |  | 3 | -.0097 | .02763 | .728 | -.0656 | .0462 |
|  |  |  | 4 | -.0497 | .03110 | .118 | -.1126 | .0132 |
|  |  | 3 | 1 | .0078 | .02854 | .787 | -.0499 | .0655 |
|  |  |  | 2 | .0097 | .02763 | .728 | -.0462 | .0656 |
|  |  |  | 4 | -.0400 | .02854 | .169 | -.0977 | .0177 |
|  |  | 4 | 1 | .0478 | .03190 | .142 | -.0168 | .1123 |
|  |  |  | 2 | .0497 | .03110 | .118 | -.0132 | .1126 |
|  |  |  | 3 | .0400 | .02854 | .169 | -.0177 | .0977 |
| d7 | LSD | 1 | 2 | .0039 | .03445 | .911 | -.0658 | .0736 |
|  |  |  | 3 | -.0031 | .03161 | .922 | -.0670 | .0608 |
|  |  |  | 4 | -.0456 | .03534 | .205 | -.1170 | .0259 |
|  |  | 2 | 1 | -.0039 | .03445 | .911 | -.0736 | .0658 |
|  |  |  | 3 | -.0070 | .03061 | .820 | -.0689 | .0549 |
|  |  |  | 4 | -.0494 | .03445 | .159 | -.1191 | .0202 |
|  |  | 3 | 1 | .0031 | .03161 | .922 | -.0608 | .0670 |
|  |  |  | 2 | .0070 | .03061 | .820 | -.0549 | .0689 |
|  |  |  | 4 | -.0424 | .03161 | .187 | -.1064 | .0215 |
|  |  | 4 | 1 | .0456 | .03534 | .205 | -.0259 | .1170 |
|  |  |  | 2 | .0494 | .03445 | .159 | -.0202 | .1191 |
|  |  |  | 3 | .0424 | .03161 | .187 | -.0215 | .1064 |
| d14 | LSD | 1 | 2 | -.0066 | .03918 | .868 | -.0858 | .0727 |
|  |  |  | 3 | -.0089 | .03595 | .806 | -.0816 | .0638 |
|  |  |  | 4 | -.0344 | .04019 | .397 | -.1157 | .0469 |
|  |  | 2 | 1 | .0066 | .03918 | .868 | -.0727 | .0858 |
|  |  |  | 3 | -.0023 | .03481 | .947 | -.0727 | .0681 |
|  |  |  | 4 | -.0279 | .03918 | .481 | -.1071 | .0514 |
|  |  | 3 | 1 | .0089 | .03595 | .806 | -.0638 | .0816 |
|  |  |  | 2 | .0023 | .03481 | .947 | -.0681 | .0727 |
|  |  |  | 4 | -.0256 | .03595 | .481 | -.0983 | .0472 |
|  |  | 4 | 1 | .0344 | .04019 | .397 | -.0469 | .1157 |
|  |  |  | 2 | .0279 | .03918 | .481 | -.0514 | .1071 |
|  |  |  | 3 | .0256 | .03595 | .481 | -.0472 | .0983 |
| d21 | LSD | 1 | 2 | -.0162 | .04527 | .722 | -.1078 | .0753 |
|  |  |  | 3 | -.0156 | .04154 | .710 | -.0996 | .0685 |
|  |  |  | 4 | .0089 | .04645 | .849 | -.0851 | .1028 |
|  |  | 2 | 1 | .0162 | .04527 | .722 | -.0753 | .1078 |
|  |  |  | 3 | .0007 | .04022 | .987 | -.0807 | .0820 |
|  |  |  | 4 | .0251 | .04527 | .582 | -.0665 | .1167 |
|  |  | 3 | 1 | .0156 | .04154 | .710 | -.0685 | .0996 |
|  |  |  | 2 | -.0007 | .04022 | .987 | -.0820 | .0807 |
|  |  |  | 4 | .0244 | .04154 | .560 | -.0596 | .1085 |
|  |  | 4 | 1 | -.0089 | .04645 | .849 | -.1028 | .0851 |
|  |  |  | 2 | -.0251 | .04527 | .582 | -.1167 | .0665 |
|  |  |  | 3 | -.0244 | .04154 | .560 | -.1085 | .0596 |
| d28 | LSD | 1 | 2 | -.0412 | .05164 | .430 | -.1457 | .0632 |
|  |  |  | 3 | -.0376 | .04739 | .433 | -.1334 | .0583 |
|  |  |  | 4 | -.0311 | .05299 | .560 | -.1383 | .0761 |
|  |  | 2 | 1 | .0412 | .05164 | .430 | -.0632 | .1457 |
|  |  |  | 3 | .0037 | .04589 | .937 | -.0891 | .0965 |
|  |  |  | 4 | .0101 | .05164 | .846 | -.0943 | .1146 |
|  |  | 3 | 1 | .0376 | .04739 | .433 | -.0583 | .1334 |
|  |  |  | 2 | -.0037 | .04589 | .937 | -.0965 | .0891 |
|  |  |  | 4 | .0064 | .04739 | .893 | -.0894 | .1023 |
|  |  | 4 | 1 | .0311 | .05299 | .560 | -.0761 | .1383 |
|  |  |  | 2 | -.0101 | .05164 | .846 | -.1146 | .0943 |
|  |  |  | 3 | -.0064 | .04739 | .893 | -.1023 | .0894 |
| d35 | LSD | 1 | 2 | .0406 | .06359 | .527 | -.0881 | .1692 |
|  |  |  | 3 | -.0304 | .05836 | .605 | -.1485 | .0876 |
|  |  |  | 4 | .0844 | .06525 | .203 | -.0475 | .2164 |
|  |  | 2 | 1 | -.0406 | .06359 | .527 | -.1692 | .0881 |
|  |  |  | 3 | -.0710 | .05651 | .216 | -.1853 | .0433 |
|  |  |  | 4 | .0439 | .06359 | .494 | -.0847 | .1725 |
|  |  | 3 | 1 | .0304 | .05836 | .605 | -.0876 | .1485 |
|  |  |  | 2 | .0710 | .05651 | .216 | -.0433 | .1853 |
|  |  |  | 4 | .1149 | .05836 | .056 | -.0032 | .2329 |
|  |  | 4 | 1 | -.0844 | .06525 | .203 | -.2164 | .0475 |
|  |  |  | 2 | -.0439 | .06359 | .494 | -.1725 | .0847 |
|  |  |  | 3 | -.1149 | .05836 | .056 | -.2329 | .0032 |

Based on observed means.

* The mean difference is significant at the .05 level.

**4. 2,3-DPG**

**A. Pairwise Comparisons between groups:**

| (I) Group | (J) Group | Mean Difference (I-J) | Std. Error | Sig.(a) | 95% Confidence Interval for Difference(a) | |
| --- | --- | --- | --- | --- | --- | --- |
|  |  |  |  |  | Upper Bound | Lower Bound |
| 1 | 2 | .705 | .571 | 1.000 | -.882 | 2.292 |
|  | 3 | .491 | .524 | 1.000 | -.965 | 1.948 |
|  | 4 | .430 | .586 | 1.000 | -1.199 | 2.058 |
| 2 | 1 | -.705 | .571 | 1.000 | -2.292 | .882 |
|  | 3 | -.214 | .507 | 1.000 | -1.624 | 1.196 |
|  | 4 | -.275 | .571 | 1.000 | -1.863 | 1.312 |
| 3 | 1 | -.491 | .524 | 1.000 | -1.948 | .965 |
|  | 2 | .214 | .507 | 1.000 | -1.196 | 1.624 |
|  | 4 | -.062 | .524 | 1.000 | -1.518 | 1.395 |
| 4 | 1 | -.430 | .586 | 1.000 | -2.058 | 1.199 |
|  | 2 | .275 | .571 | 1.000 | -1.312 | 1.863 |
|  | 3 | .062 | .524 | 1.000 | -1.395 | 1.518 |

Based on estimated marginal means

a Adjustment for multiple comparisons: Bonferroni.

**B. Multiple Comparisons at the same time point among groups:**

| Dependent Variable |  | (I) Group | (J) Group | Mean Difference (I-J) | Std. Error | Sig. | 95% Confidence Interval | |
| --- | --- | --- | --- | --- | --- | --- | --- | --- |
|  |  |  |  |  |  |  | Upper Bound | Lower Bound |
| d0 | LSD | 1 | 2 | 1.0612 | .97343 | .282 | -.9077 | 3.0302 |
|  |  |  | 3 | .8062 | .89328 | .372 | -1.0007 | 2.6130 |
|  |  |  | 4 | -.2502 | .99872 | .803 | -2.2703 | 1.7699 |
|  |  | 2 | 1 | -1.0612 | .97343 | .282 | -3.0302 | .9077 |
|  |  |  | 3 | -.2551 | .86491 | .770 | -2.0045 | 1.4944 |
|  |  |  | 4 | -1.3114 | .97343 | .186 | -3.2804 | .6575 |
|  |  | 3 | 1 | -.8062 | .89328 | .372 | -2.6130 | 1.0007 |
|  |  |  | 2 | .2551 | .86491 | .770 | -1.4944 | 2.0045 |
|  |  |  | 4 | -1.0564 | .89328 | .244 | -2.8632 | .7505 |
|  |  | 4 | 1 | .2502 | .99872 | .803 | -1.7699 | 2.2703 |
|  |  |  | 2 | 1.3114 | .97343 | .186 | -.6575 | 3.2804 |
|  |  |  | 3 | 1.0564 | .89328 | .244 | -.7505 | 2.8632 |
| d7 | LSD | 1 | 2 | 1.2495 | .70595 | .085 | -.1784 | 2.6774 |
|  |  |  | 3 | .7930 | .64782 | .228 | -.5174 | 2.1033 |
|  |  |  | 4 | .7205 | .72428 | .326 | -.7445 | 2.1855 |
|  |  | 2 | 1 | -1.2495 | .70595 | .085 | -2.6774 | .1784 |
|  |  |  | 3 | -.4565 | .62725 | .471 | -1.7253 | .8122 |
|  |  |  | 4 | -.5290 | .70595 | .458 | -1.9569 | .8989 |
|  |  | 3 | 1 | -.7930 | .64782 | .228 | -2.1033 | .5174 |
|  |  |  | 2 | .4565 | .62725 | .471 | -.8122 | 1.7253 |
|  |  |  | 4 | -.0725 | .64782 | .912 | -1.3828 | 1.2379 |
|  |  | 4 | 1 | -.7205 | .72428 | .326 | -2.1855 | .7445 |
|  |  |  | 2 | .5290 | .70595 | .458 | -.8989 | 1.9569 |
|  |  |  | 3 | .0725 | .64782 | .912 | -1.2379 | 1.3828 |
| d14 | LSD | 1 | 2 | .7512 | .58994 | .210 | -.4421 | 1.9445 |
|  |  |  | 3 | .4266 | .54137 | .435 | -.6684 | 1.5217 |
|  |  |  | 4 | .7136 | .60527 | .246 | -.5107 | 1.9378 |
|  |  | 2 | 1 | -.7512 | .58994 | .210 | -1.9445 | .4421 |
|  |  |  | 3 | -.3245 | .52418 | .539 | -1.3848 | .7357 |
|  |  |  | 4 | -.0376 | .58994 | .949 | -1.2309 | 1.1556 |
|  |  | 3 | 1 | -.4266 | .54137 | .435 | -1.5217 | .6684 |
|  |  |  | 2 | .3245 | .52418 | .539 | -.7357 | 1.3848 |
|  |  |  | 4 | .2869 | .54137 | .599 | -.8081 | 1.3819 |
|  |  | 4 | 1 | -.7136 | .60527 | .246 | -1.9378 | .5107 |
|  |  |  | 2 | .0376 | .58994 | .949 | -1.1556 | 1.2309 |
|  |  |  | 3 | -.2869 | .54137 | .599 | -1.3819 | .8081 |
| d21 | LSD | 1 | 2 | .5504 | .50583 | .283 | -.4727 | 1.5735 |
|  |  |  | 3 | .3908 | .46418 | .405 | -.5481 | 1.3297 |
|  |  |  | 4 | .6886 | .51897 | .192 | -.3611 | 1.7383 |
|  |  | 2 | 1 | -.5504 | .50583 | .283 | -1.5735 | .4727 |
|  |  |  | 3 | -.1596 | .44944 | .724 | -1.0687 | .7494 |
|  |  |  | 4 | .1382 | .50583 | .786 | -.8850 | 1.1613 |
|  |  | 3 | 1 | -.3908 | .46418 | .405 | -1.3297 | .5481 |
|  |  |  | 2 | .1596 | .44944 | .724 | -.7494 | 1.0687 |
|  |  |  | 4 | .2978 | .46418 | .525 | -.6411 | 1.2367 |
|  |  | 4 | 1 | -.6886 | .51897 | .192 | -1.7383 | .3611 |
|  |  |  | 2 | -.1382 | .50583 | .786 | -1.1613 | .8850 |
|  |  |  | 3 | -.2978 | .46418 | .525 | -1.2367 | .6411 |
| d28 | LSD | 1 | 2 | .3950 | .42688 | .360 | -.4684 | 1.2585 |
|  |  |  | 3 | .3557 | .39173 | .369 | -.4367 | 1.1480 |
|  |  |  | 4 | .4606 | .43797 | .299 | -.4253 | 1.3464 |
|  |  | 2 | 1 | -.3950 | .42688 | .360 | -1.2585 | .4684 |
|  |  |  | 3 | -.0394 | .37929 | .918 | -.8065 | .7278 |
|  |  |  | 4 | .0655 | .42688 | .879 | -.7979 | .9290 |
|  |  | 3 | 1 | -.3557 | .39173 | .369 | -1.1480 | .4367 |
|  |  |  | 2 | .0394 | .37929 | .918 | -.7278 | .8065 |
|  |  |  | 4 | .1049 | .39173 | .790 | -.6874 | .8972 |
|  |  | 4 | 1 | -.4606 | .43797 | .299 | -1.3464 | .4253 |
|  |  |  | 2 | -.0655 | .42688 | .879 | -.9290 | .7979 |
|  |  |  | 3 | -.1049 | .39173 | .790 | -.8972 | .6874 |
| d35 | LSD | 1 | 2 | .2235 | .35238 | .530 | -.4892 | .9363 |
|  |  |  | 3 | .1756 | .32337 | .590 | -.4785 | .8297 |
|  |  |  | 4 | .2453 | .36154 | .501 | -.4859 | .9766 |
|  |  | 2 | 1 | -.2235 | .35238 | .530 | -.9363 | .4892 |
|  |  |  | 3 | -.0479 | .31310 | .879 | -.6812 | .5854 |
|  |  |  | 4 | .0218 | .35238 | .951 | -.6910 | .7346 |
|  |  | 3 | 1 | -.1756 | .32337 | .590 | -.8297 | .4785 |
|  |  |  | 2 | .0479 | .31310 | .879 | -.5854 | .6812 |
|  |  |  | 4 | .0697 | .32337 | .830 | -.5843 | .7238 |
|  |  | 4 | 1 | -.2453 | .36154 | .501 | -.9766 | .4859 |
|  |  |  | 2 | -.0218 | .35238 | .951 | -.7346 | .6910 |
|  |  |  | 3 | -.0697 | .32337 | .830 | -.7238 | .5843 |

Based on observed means.

* The mean difference is significant at the .05 level.

**5. EMP:**

**A.Pairwise Comparisons between groups:**

| (I) Group | (J) Group | Mean Difference (I-J) | Std. Error | Sig.(a) | 95% Confidence Interval for Difference(a) | |
| --- | --- | --- | --- | --- | --- | --- |
|  |  |  |  |  | Upper Bound | Lower Bound |
| 1 | 2 | 1.200 | 2.098 | 1.000 | -4.687 | 7.088 |
|  | 3 | -2.762 | 1.876 | .903 | -8.028 | 2.504 |
|  | 4 | 4.441 | 2.098 | .251 | -1.446 | 10.329 |
| 2 | 1 | -1.200 | 2.098 | 1.000 | -7.088 | 4.687 |
|  | 3 | -3.962 | 1.700 | .156 | -8.735 | .810 |
|  | 4 | 3.241 | 1.942 | .628 | -2.210 | 8.692 |
| 3 | 1 | 2.762 | 1.876 | .903 | -2.504 | 8.028 |
|  | 2 | 3.962 | 1.700 | .156 | -.810 | 8.735 |
|  | 4 | 7.203(*) | 1.700 | .001 | 2.431 | 11.976 |
| 4 | 1 | -4.441 | 2.098 | .251 | -10.329 | 1.446 |
|  | 2 | -3.241 | 1.942 | .628 | -8.692 | 2.210 |
|  | 3 | -7.203(*) | 1.700 | .001 | -11.976 | -2.431 |

Based on estimated marginal means

* The mean difference is significant at the .05 level.

a Adjustment for multiple comparisons: Bonferroni.

**B. Multiple Comparisons at the same time point among groups:**

| Dependent Variable |  | (I) Group | (J) Group | Mean Difference (I-J) | Std. Error | Sig. | 95% Confidence Interval | |
| --- | --- | --- | --- | --- | --- | --- | --- | --- |
|  |  |  |  |  |  |  | Upper Bound | Lower Bound |
| d0 | LSD | 1 | 2 | -.7050 | 2.27785 | .759 | -5.3393 | 3.9293 |
|  |  |  | 3 | -3.2953 | 2.03737 | .115 | -7.4404 | .8497 |
|  |  |  | 4 | .3300 | 2.27785 | .886 | -4.3043 | 4.9643 |
|  |  | 2 | 1 | .7050 | 2.27785 | .759 | -3.9293 | 5.3393 |
|  |  |  | 3 | -2.5903 | 1.84653 | .170 | -6.3471 | 1.1665 |
|  |  |  | 4 | 1.0350 | 2.10888 | .627 | -3.2556 | 5.3256 |
|  |  | 3 | 1 | 3.2953 | 2.03737 | .115 | -.8497 | 7.4404 |
|  |  |  | 2 | 2.5903 | 1.84653 | .170 | -1.1665 | 6.3471 |
|  |  |  | 4 | 3.6253 | 1.84653 | .058 | -.1315 | 7.3821 |
|  |  | 4 | 1 | -.3300 | 2.27785 | .886 | -4.9643 | 4.3043 |
|  |  |  | 2 | -1.0350 | 2.10888 | .627 | -5.3256 | 3.2556 |
|  |  |  | 3 | -3.6253 | 1.84653 | .058 | -7.3821 | .1315 |
| d7 | LSD | 1 | 2 | -.0092 | 2.07477 | .997 | -4.2303 | 4.2120 |
|  |  |  | 3 | -3.2217 | 1.85573 | .092 | -6.9972 | .5538 |
|  |  |  | 4 | 1.0083 | 2.07477 | .630 | -3.2128 | 5.2295 |
|  |  | 2 | 1 | .0092 | 2.07477 | .997 | -4.2120 | 4.2303 |
|  |  |  | 3 | -3.2125 | 1.68190 | .065 | -6.6343 | .2093 |
|  |  |  | 4 | 1.0175 | 1.92086 | .600 | -2.8905 | 4.9255 |
|  |  | 3 | 1 | 3.2217 | 1.85573 | .092 | -.5538 | 6.9972 |
|  |  |  | 2 | 3.2125 | 1.68190 | .065 | -.2093 | 6.6343 |
|  |  |  | 4 | 4.2300(*) | 1.68190 | .017 | .8082 | 7.6518 |
|  |  | 4 | 1 | -1.0083 | 2.07477 | .630 | -5.2295 | 3.2128 |
|  |  |  | 2 | -1.0175 | 1.92086 | .600 | -4.9255 | 2.8905 |
|  |  |  | 3 | -4.2300(*) | 1.68190 | .017 | -7.6518 | -.8082 |
| d14 | LSD | 1 | 2 | 1.6358 | 2.32990 | .488 | -3.1044 | 6.3760 |
|  |  |  | 3 | -1.9830 | 2.08392 | .348 | -6.2228 | 2.2568 |
|  |  |  | 4 | 3.0383 | 2.32990 | .201 | -1.7019 | 7.7785 |
|  |  | 2 | 1 | -1.6358 | 2.32990 | .488 | -6.3760 | 3.1044 |
|  |  |  | 3 | -3.6188 | 1.88871 | .064 | -7.4615 | .2238 |
|  |  |  | 4 | 1.4025 | 2.15706 | .520 | -2.9861 | 5.7911 |
|  |  | 3 | 1 | 1.9830 | 2.08392 | .348 | -2.2568 | 6.2228 |
|  |  |  | 2 | 3.6188 | 1.88871 | .064 | -.2238 | 7.4615 |
|  |  |  | 4 | 5.0213(*) | 1.88871 | .012 | 1.1787 | 8.8640 |
|  |  | 4 | 1 | -3.0383 | 2.32990 | .201 | -7.7785 | 1.7019 |
|  |  |  | 2 | -1.4025 | 2.15706 | .520 | -5.7911 | 2.9861 |
|  |  |  | 3 | -5.0213(*) | 1.88871 | .012 | -8.8640 | -1.1787 |
| d21 | LSD | 1 | 2 | 1.5042 | 2.47588 | .548 | -3.5330 | 6.5414 |
|  |  |  | 3 | -2.4860 | 2.21449 | .270 | -6.9914 | 2.0194 |
|  |  |  | 4 | 4.6754 | 2.47588 | .068 | -.3618 | 9.7126 |
|  |  | 2 | 1 | -1.5042 | 2.47588 | .548 | -6.5414 | 3.5330 |
|  |  |  | 3 | -3.9902 | 2.00706 | .055 | -8.0736 | .0932 |
|  |  |  | 4 | 3.1713 | 2.29222 | .176 | -1.4923 | 7.8348 |
|  |  | 3 | 1 | 2.4860 | 2.21449 | .270 | -2.0194 | 6.9914 |
|  |  |  | 2 | 3.9902 | 2.00706 | .055 | -.0932 | 8.0736 |
|  |  |  | 4 | 7.1614(*) | 2.00706 | .001 | 3.0780 | 11.2448 |
|  |  | 4 | 1 | -4.6754 | 2.47588 | .068 | -9.7126 | .3618 |
|  |  |  | 2 | -3.1713 | 2.29222 | .176 | -7.8348 | 1.4923 |
|  |  |  | 3 | -7.1614(*) | 2.00706 | .001 | -11.2448 | -3.0780 |
| d28 | LSD | 1 | 2 | 3.3167 | 2.37473 | .172 | -1.5148 | 8.1481 |
|  |  |  | 3 | -2.2977 | 2.12402 | .287 | -6.6190 | 2.0237 |
|  |  |  | 4 | 6.8217(*) | 2.37473 | .007 | 1.9902 | 11.6531 |
|  |  | 2 | 1 | -3.3167 | 2.37473 | .172 | -8.1481 | 1.5148 |
|  |  |  | 3 | -5.6143(*) | 1.92506 | .006 | -9.5309 | -1.6978 |
|  |  |  | 4 | 3.5050 | 2.19857 | .120 | -.9680 | 7.9780 |
|  |  | 3 | 1 | 2.2977 | 2.12402 | .287 | -2.0237 | 6.6190 |
|  |  |  | 2 | 5.6143(*) | 1.92506 | .006 | 1.6978 | 9.5309 |
|  |  |  | 4 | 9.1193(*) | 1.92506 | .000 | 5.2028 | 13.0359 |
|  |  | 4 | 1 | -6.8217(*) | 2.37473 | .007 | -11.6531 | -1.9902 |
|  |  |  | 2 | -3.5050 | 2.19857 | .120 | -7.9780 | .9680 |
|  |  |  | 3 | -9.1193(*) | 1.92506 | .000 | -13.0359 | -5.2028 |
| d35 | LSD | 1 | 2 | 1.4600 | 2.95270 | .624 | -4.5473 | 7.4673 |
|  |  |  | 3 | -3.2870 | 2.64098 | .222 | -8.6601 | 2.0861 |
|  |  |  | 4 | 10.7750(*) | 2.95270 | .001 | 4.7677 | 16.7823 |
|  |  | 2 | 1 | -1.4600 | 2.95270 | .624 | -7.4673 | 4.5473 |
|  |  |  | 3 | -4.7470 | 2.39359 | .056 | -9.6168 | .1228 |
|  |  |  | 4 | 9.3150(*) | 2.73367 | .002 | 3.7533 | 14.8767 |
|  |  | 3 | 1 | 3.2870 | 2.64098 | .222 | -2.0861 | 8.6601 |
|  |  |  | 2 | 4.7470 | 2.39359 | .056 | -.1228 | 9.6168 |
|  |  |  | 4 | 14.0620(*) | 2.39359 | .000 | 9.1922 | 18.9318 |
|  |  | 4 | 1 | -10.7750(*) | 2.95270 | .001 | -16.7823 | -4.7677 |
|  |  |  | 2 | -9.3150(*) | 2.73367 | .002 | -14.8767 | -3.7533 |
|  |  |  | 3 | -14.0620(*) | 2.39359 | .000 | -18.9318 | -9.1922 |

Based on observed means.

* The mean difference is significant at the .05 level.

**6. Hemolysis:**

**A.Pairwise Comparisons between groups:**

| (I) Group | (J) Group | Mean Difference (I-J) | Std. Error | Sig.(a) | 95% Confidence Interval for Difference(a) | |
| --- | --- | --- | --- | --- | --- | --- |
|  |  |  |  |  | Upper Bound | Lower Bound |
| 1 | 2 | -.044 | .016 | .053 | -.088 | .000 |
|  | 3 | -.033 | .015 | .169 | -.074 | .007 |
|  | 4 | .006 | .016 | 1.000 | -.039 | .052 |
| 2 | 1 | .044 | .016 | .053 | .000 | .088 |
|  | 3 | .011 | .014 | 1.000 | -.029 | .050 |
|  | 4 | .050(*) | .016 | .018 | .006 | .095 |
| 3 | 1 | .033 | .015 | .169 | -.007 | .074 |
|  | 2 | -.011 | .014 | 1.000 | -.050 | .029 |
|  | 4 | .040 | .015 | .058 | -.001 | .080 |
| 4 | 1 | -.006 | .016 | 1.000 | -.052 | .039 |
|  | 2 | -.050(*) | .016 | .018 | -.095 | -.006 |
|  | 3 | -.040 | .015 | .058 | -.080 | .001 |

**B. Multiple Comparisons at the same time point among groups:**

| Dependent Variable |  | (I) Group | (J) Group | Mean Difference (I-J) | Std. Error | Sig. | 95% Confidence Interval | |
| --- | --- | --- | --- | --- | --- | --- | --- | --- |
|  |  |  |  |  |  |  | Upper Bound | Lower Bound |
| d0 | LSD | 1 | 2 | -.0214 | .00891 | .128 | -.0461 | .0034 |
|  |  |  | 3 | -.0102 | .00818 | .219 | -.0267 | .0063 |
|  |  |  | 4 | .0046 | .00914 | .621 | -.0139 | .0231 |
|  |  | 2 | 1 | .0214 | .00891 | .128 | -.0034 | .0461 |
|  |  |  | 3 | .0112 | .00792 | .167 | -.0049 | .0272 |
|  |  |  | 4 | .0259(*) | .00891 | .006 | .0079 | .0440 |
|  |  | 3 | 1 | .0102 | .00818 | .219 | -.0063 | .0267 |
|  |  |  | 2 | -.0112 | .00792 | .167 | -.0272 | .0049 |
|  |  |  | 4 | .0148 | .00818 | .079 | -.0018 | .0313 |
|  |  | 4 | 1 | -.0046 | .00914 | .621 | -.0231 | .0139 |
|  |  |  | 2 | -.0259(*) | .00891 | .006 | -.0440 | -.0079 |
|  |  |  | 3 | -.0148 | .00818 | .079 | -.0313 | .0018 |
| d7 | LSD | 1 | 2 | -.0213 | .01008 | .245 | -.0493 | .0067 |
|  |  |  | 3 | -.0134 | .00925 | .154 | -.0322 | .0053 |
|  |  |  | 4 | .0082 | .01034 | .431 | -.0127 | .0291 |
|  |  | 2 | 1 | .0213 | .01008 | .245 | -.0067 | .0493 |
|  |  |  | 3 | .0079 | .00895 | .385 | -.0102 | .0260 |
|  |  |  | 4 | .0296(*) | .01008 | .006 | .0092 | .0499 |
|  |  | 3 | 1 | .0134 | .00925 | .154 | -.0053 | .0322 |
|  |  |  | 2 | -.0079 | .00895 | .385 | -.0260 | .0102 |
|  |  |  | 4 | .0217(*) | .00925 | .024 | .0030 | .0404 |
|  |  | 4 | 1 | -.0082 | .01034 | .431 | -.0291 | .0127 |
|  |  |  | 2 | -.0296(*) | .01008 | .006 | -.0499 | -.0092 |
|  |  |  | 3 | -.0217(*) | .00925 | .024 | -.0404 | -.0030 |
| d14 | LSD | 1 | 2 | -.0309(*) | .01424 | .036 | -.0597 | -.0021 |
|  |  |  | 3 | -.0261 | .01307 | .053 | -.0525 | .0003 |
|  |  |  | 4 | .0118 | .01461 | .425 | -.0178 | .0413 |
|  |  | 2 | 1 | .0309(*) | .01424 | .036 | .0021 | .0597 |
|  |  |  | 3 | .0048 | .01265 | .705 | -.0208 | .0304 |
|  |  |  | 4 | .0427(*) | .01424 | .005 | .0139 | .0715 |
|  |  | 3 | 1 | .0261 | .01307 | .053 | -.0003 | .0525 |
|  |  |  | 2 | -.0048 | .01265 | .705 | -.0304 | .0208 |
|  |  |  | 4 | .0379(*) | .01307 | .006 | .0114 | .0643 |
|  |  | 4 | 1 | -.0118 | .01461 | .425 | -.0413 | .0178 |
|  |  |  | 2 | -.0427(*) | .01424 | .005 | -.0715 | -.0139 |
|  |  |  | 3 | -.0379(*) | .01307 | .006 | -.0643 | -.0114 |
| d21 | LSD | 1 | 2 | -.0457(*) | .01781 | .014 | -.0817 | -.0096 |
|  |  |  | 3 | -.0422(*) | .01635 | .014 | -.0753 | -.0091 |
|  |  |  | 4 | .0123 | .01828 | .504 | -.0246 | .0493 |
|  |  | 2 | 1 | .0457(*) | .01781 | .014 | .0096 | .0817 |
|  |  |  | 3 | .0035 | .01583 | .827 | -.0285 | .0355 |
|  |  |  | 4 | .0580(*) | .01781 | .002 | .0220 | .0940 |
|  |  | 3 | 1 | .0422(*) | .01635 | .014 | .0091 | .0753 |
|  |  |  | 2 | -.0035 | .01583 | .827 | -.0355 | .0285 |
|  |  |  | 4 | .0545(*) | .01635 | .002 | .0215 | .0876 |
|  |  | 4 | 1 | -.0123 | .01828 | .504 | -.0493 | .0246 |
|  |  |  | 2 | -.0580(*) | .01781 | .002 | -.0940 | -.0220 |
|  |  |  | 3 | -.0545(*) | .01635 | .002 | -.0876 | -.0215 |
| d28 | LSD | 1 | 2 | -.0622(*) | .02143 | .006 | -.1056 | -.0188 |
|  |  |  | 3 | -.0523(*) | .01967 | .011 | -.0921 | -.0126 |
|  |  |  | 4 | .0050 | .02199 | .820 | -.0394 | .0495 |
|  |  | 2 | 1 | .0622(*) | .02143 | .006 | .0188 | .1056 |
|  |  |  | 3 | .0099 | .01904 | .607 | -.0287 | .0484 |
|  |  |  | 4 | .0672(*) | .02143 | .003 | .0239 | .1106 |
|  |  | 3 | 1 | .0523(*) | .01967 | .011 | .0126 | .0921 |
|  |  |  | 2 | -.0099 | .01904 | .607 | -.0484 | .0287 |
|  |  |  | 4 | .0574(*) | .01967 | .006 | .0176 | .0972 |
|  |  | 4 | 1 | -.0050 | .02199 | .820 | -.0495 | .0394 |
|  |  |  | 2 | -.0672(*) | .02143 | .003 | -.1106 | -.0239 |
|  |  |  | 3 | -.0574(*) | .01967 | .006 | -.0972 | -.0176 |
| d35 | LSD | 1 | 2 | -.0816(*) | .02928 | .008 | -.1409 | -.0224 |
|  |  |  | 3 | -.0555(*) | .02687 | .046 | -.1099 | -.0012 |
|  |  |  | 4 | -.0030 | .03004 | .922 | -.0637 | .0578 |
|  |  | 2 | 1 | .0816(*) | .02928 | .008 | .0224 | .1409 |
|  |  |  | 3 | .0261 | .02602 | .321 | -.0265 | .0788 |
|  |  |  | 4 | .0787(*) | .02928 | .011 | .0195 | .1379 |
|  |  | 3 | 1 | .0555(*) | .02687 | .046 | .0012 | .1099 |
|  |  |  | 2 | -.0261 | .02602 | .321 | -.0788 | .0265 |
|  |  |  | 4 | .0525 | .02687 | .058 | -.0018 | .1069 |
|  |  | 4 | 1 | .0030 | .03004 | .922 | -.0578 | .0637 |
|  |  |  | 2 | -.0787(*) | .02928 | .011 | -.1379 | -.0195 |
|  |  |  | 3 | -.0525 | .02687 | .058 | -.1069 | .0018 |

Based on observed means.

* The mean difference is significant at the .05 level.
