# Supplementary material for: Erythrocyte Concentrates Recovered from Under-Collected Whole Blood: Experimental and Clinical Results
Source: PLoS One. 2015 Feb 23;10(2):e0117928. doi: 10.1371/journal.pone.0117928 (PMC4338272; doi:10.1371/journal.pone.0117928)
Supplement: S2 File — (PDF) [file pone.0117928.s005.pdf]

Adverse Transfusion Events (ATEs)

1. Incidents

Incident is a deviation from standard operating procedure or hospital policy during the transfusion process.

Examples:

- Mislabelled specimens
- Blood component ordered for the wrong patient
- Wrong component ordered for patient
- Laboratory error
- Component administered to wrong patient
- Incorrect equipment used for the transfusion
- Dispensing of expired or unsuitable component

1.1 **Complete incident:** Incident which was recognised after transfusion started or occurred during transfusion.

1.2 **Near miss incident:** Incident which was recognised before transfusion started.

2. Complications

Complications are adverse reactions to or adverse effects of the transfused blood components.

2.1. Transfusion-transmitted infections (TTI)

2.2 Immune Complications of Transfusion (ICT)

2.3 Cardiovascular and Metabolic Complications of Transfusion (CMCT)

2.4 Previously Unknown Complication of Transfusion (PUCT)

(see next pages)

2.1. Transfusion-transmitted infections (TTI)

Infectious agent	Definition
**2.1.1 Viral infection (TTVI)**	Following investigation, the recipient has evidence of infection post-transfusion and no clinical or laboratory evidence of infection prior to transfusion<br>and **either,** at least one component received by the infected recipient was donated by a donor who had evidence of the same infection,**or,** at least one component received by the infected recipient was shown to have been contaminated with the virus.
**2.1.2 Bacterial infection (TTBI)**	*TTBI should be clinically suspected if:*<br>• fever $\geq$ 39°C or a change of $\geq$ 2°C from pretransfusion value and<br>• rigors and<br>• tachycardia $\geq$ 120 beats / min or a change of $\geq$ 40 beats / min from pretransfusion value or a rise or drop of 30 mm Hg in systolic blood pressure<br>within 4 hours of transfusion are present.<br><br>*Possible TTBI:*<br>• detection of bacteria by approved techniques in the transfused blood component but not in the recipient`s blood or<br>• detection of bacteria in the recipient`s blood following transfusion but not in the transfused blood component and no other reasons are ascertainable for the positive blood culture.<br><br>*Confirmed TTBI:* detection of the same bacterial strain in the recipient's blood and in the transfused blood product by approved techniques.
**2.1.3 Parasites (TTPI)**	Detection of the same parasite in the recipient's blood and parasite or specific antibodies in the donor blood

2.2 Immune Complications of Transfusion (ICT)

Complication	Definition
2.2.1 Haemolytic transfusion reaction (HTR)	HTR is clinically suspected if one or more of :<br>• fever and a variety of other symptoms (including dyspnea, hypotension, tachycardia, flank or back pain, etc)<br>• inadequate rise of post-transfusion hemoglobin level<br>• drop in haemoglobin level ($\geq$ 2g/dl within 24 hours)<br>• rise in LDH ($\geq$ 50% within 24 hours)<br>• rise in bilirubin, haemoglobinaemia, decrease in haptoglobin<br>is present in a temporal association with transfusion.<br><br>HTR is confirmed by a<br>• a positive direct antiglobulin test and<br>• a positive erythrocyte cross-match.<br>---<br>(Two *subtypes* of HTR are clinically distinguished:<br>• *Acute HTR*: occurrence within 24 hours of transfusion<br>• *Delayed HTR*: occurrence between 1 – 28 days after transfusion)
2.2.2 Febrile non-haemolytic transfusion reaction (FNHTR) (Transfusion-related discomfort)	One or more of:<br>• fever ($\geq$ 38°C or a change of $\geq$1 ^{0}C from pretransfusion value)<br>• chills<br>• cold<br>• rigor<br>• (other symptoms of discomfort)<br>during or within 4 hours following transfusion without any other cause such as HTR or TTBI.
2.2.3 Transfusion-related acute lung injury (TRALI)	• acute respiratory distress <u>and</u><br>• bilateral lung infiltrations in the chest radiograph <u>and</u><br>• occurrence during or within 6 hours of the completion of the transfusion <u>and</u><br>• no evidence of transfusion-associated circulatory overload<br>---<br>(*Subtypes*, determination is not necessary for diagnosis):<br>• *Immune (antibody-mediated) TRALI* is confirmed by the detection of leucocyte (HLA and HNA) antibodies in the donor's or recipient's blood and a corresponding leucocyte antigen typing or a positive granulocyte cross-match.<br>• *Non-immune (not antibody-mediated) TRALI:* No leucocyte antibodies detetable by reference laboratories using accepted techniques

Complication	Definition
2.2.4 Transfusion-induced graft versus host disease (TI-GVHD)	Fever, rash, liver dysfunction, diarrhea and cytopenia 1 – 6 weeks following transfusion with no other apparent cause.<br>GVHD is confirmed by GVHD-typical biopsy and by genetic analysis to show chimerism of recipient and donor lymphocytes
2.2.5 Posttransfusion purpura (PTP)	Purpura and thrombocytopenia within 12 days after transfusion.<br>PTP is confirmed by the detection of platelet-specific anti-bodies (usually anti-HPA-1a) in the recipient's blood and by a corresponding platelet antigen typing of the donor or by a positive platelet cross-match
2.2.6 Allergic reaction	One or more of:<br>• rash,<br>• allergic dyspnea (stridor, cyanosis, wheezing)<br>• angioedema<br>• generalized pruritus<br>• urticaria<br>without hypotension during or within 24 hours of transfusion
2.2.7 Anaphylactoid reaction	Allergic reaction with hypotension (drop in systolic blood pressure by $\geq$ 30 mm Hg) during or within 24 hours of transfusion
2.2.8 Anaphylactic shock	Intractable hypotension or shock with loss of consciousness during transfusion, and without any indication of other cause
2.2.9 Alloimmunization	Formation of alloantibodies to RBC, HLA, HPA and HNA antigens which were not detectable pretransfusion.
2.2.10 Transfusion-associated autoimmune haemolytic anaemia (TA-AIHA)	Haemolysis-related symptoms (pallor, tachycardia, hyperventilation etc.) in a temporal association with transfusion.<br>TA-AIHA is confirmed by a drop in haemoglobin level, a positive direct antiglobulin test and an eluate revealing an erythrocyte autoantibody which was not present in the recipient`s blood pretransfusion.

2.3 Cardiovascular and Metabolic Complications of Transfusion (CMCT)

Complication	Definition
2.3.1 Transfusion-associated circulatory overload (TACO)	Respiratory distress, tachycardia, increased blood pressure, typical signs of cardiogenic lung oedema in the chest x-ray, evidence of a positive fluid balance and / or a known compromised cardiac status during or within 12 hours after transfusion.
2.3.2 Transfusion-associated dyspnea (TAD)	Respiratory distress in temporal association with blood transfusion with no evidence of TRALI, allergic dyspnea or TACO.
2.3.3 Hypothermia	Decrease of body temperature associated with transfusion resulting in dyspnea, hypotension and / or cardiac dysfunction
2.3.4 Hyperkalaemia	Abnormal increase of the potassium level associated with transfusion resulting in cardiac arrhythmias and / or dysfunction
2.3.5 Hypocalcemia	Abnormal decrease of the calcium level associated with transfusion resulting in carpopedal spasm and/or cardiac arrhythmias and / or dysfunction
2.3.6 Haemosiderosis	Iron overload as indicated by laboratory findings or biopsy due to chronic transfusion which can result in organ injury (heart, liver, lung and / or endocrine glands).
2.3.7 Hypotension	Drop in systolic blood pressure by ≥ 30 mm Hg during or within 4 hours of the completion of the transfusion and no evidence of other complications.
2.3.8 Hypertension	Rise in systolic blood pressure by ≥ 30 mm Hg during or within 4 hours of the completion of the transfusion and no evidence of other complications.

2.4 Previously Unknown Complication of Transfusion (PUCT)

Occurrence of an adverse effect or reaction temporally related to transfusion, which cannot be attributed to the already defined side effects (2.1- 2.3) and with no risk factor other than transfusion.
